# Variance of Gene Expression Identifies Altered Network Constraints in Neurological Disease

**DOI:** 10.1371/journal.pgen.1002207

**Published:** 2011-08-11

**Authors:** Jessica C. Mar, Nicholas A. Matigian, Alan Mackay-Sim, George D. Mellick, Carolyn M. Sue, Peter A. Silburn, John J. McGrath, John Quackenbush, Christine A. Wells

**Affiliations:** 1 Department of Biostatistics, Harvard School of Public Health, Boston, United States of America; 2 Department of Biostatistics and Computational Biology, Dana-Farber Cancer Institute, Boston, United States of America; 3 National Centre for Adult Stem Cell Research, Eskitis Institute for Cell and Molecular Therapies, Griffith University, Brisbane, Australia; 4 Institute for Molecular Biosciences, The University of Queensland, Brisbane, Australia; 5 Department of Neurogenetics, Kolling Institute for Medical Research, Royal North Shore Hospital and University of Sydney, Sydney, Australia; 6 The University of Queensland Centre for Clinical Research, Brisbane, Australia; 7 Queensland Brain Institute, The University of Queensland, Brisbane, Australia; 8 Department of Psychiatry, The University of Queensland, Brisbane, Australia; 9 Queensland Centre for Mental Health Research, The Park Centre for Mental Health, Brisbane, Australia; 10 Australian Institute for Bioengineering and Nanotechnology, The University of Queensland, Brisbane, Australia; Georgia Institute of Technology, United States of America

## Abstract

Gene expression analysis has become a ubiquitous tool for studying a wide range of human diseases. In a typical analysis we compare distinct phenotypic groups and attempt to identify genes that are, on average, significantly different between them. Here we describe an innovative approach to the analysis of gene expression data, one that identifies differences in expression variance between groups as an informative metric of the group phenotype. We find that genes with different expression variance profiles are not randomly distributed across cell signaling networks. Genes with low-expression variance, or higher constraint, are significantly more connected to other network members and tend to function as core members of signal transduction pathways. Genes with higher expression variance have fewer network connections and also tend to sit on the periphery of the cell. Using neural stem cells derived from patients suffering from Schizophrenia (SZ), Parkinson's disease (PD), and a healthy control group, we find marked differences in expression variance in cell signaling pathways that shed new light on potential mechanisms associated with these diverse neurological disorders. In particular, we find that expression variance of core networks in the SZ patient group was considerably constrained, while in contrast the PD patient group demonstrated much greater variance than expected. One hypothesis is that diminished variance in SZ patients corresponds to an increased degree of constraint in these pathways and a corresponding reduction in robustness of the stem cell networks. These results underscore the role that variation plays in biological systems and suggest that analysis of expression variance is far more important in disease than previously recognized. Furthermore, modeling patterns of variability in gene expression could fundamentally alter the way in which we think about how cellular networks are affected by disease processes.

## Author Summary

Genes are a repository of information that provides the framework for cellular processes, with the flow of information from gene (DNA) to phenotype via an intermediate molecule—the messenger RNA. We understand that sequence variations in a gene may lead to phenotypic variations, but less well understood is how variation in the information flow itself might also impact on phenotype. In this study we demonstrated that disease phenotypes were correlated with expression variance. A change in expression variance might infer that the genetic networks representing information flow were less robust—surprisingly, we found that too little and too much variance were equally detrimental in the context of neurological disease.

## Introduction

In studying biological systems, we tend to think of groups as being defined by specific, measurable parameters, and of the important differences between those groups as being defined by a significant average difference in those parameters. Much of the language we use in describing biological systems is based on this bias and we talk about genes being expressed in a tissue at a particular level, or about differences in gene expression between groups reflecting the mechanism driving their phenotypic differences.

This view of biological systems has been extremely useful in that nearly all of our understanding of biological systems is based on interpreting average behavior. Variance in this context is only used as a measure of the significance of those mean differences (through the use of statistical measures such as a t-test or ANOVA). Rarely has the variability across a population been considered in the analysis of transcriptional differences between populations. Arguably, variance has been largely ignored because it has been considered solely in the context of experimental reproducibility, and therefore something that must be reduced. This was a reasonable bias in the early days of microarrays, but the robustness and reproducibility of the current generation of array platforms [1] allows us to look at additional drivers of variance in gene expression studies. Increasingly there is evidence that biological sources of variation may play an important role in determining cellular and organismal phenotypes [2]-[8], as well as in helping to explain a wide range of biological phenomena ranging from reduced penetrance [9], [10] to evolutionary fitness [11].

The direct link between genetics and reduced penetrance, expression variability and phenotype was elegantly demonstrated in *C. elegans* by Raj and colleagues, who showed that variation in the number of transcripts expressed in any individual cell, as well as the number of cells expressing the transcript of interest, directly influenced the development of the worm's intestine [6]. Mutations in key developmental transcription factors affected not just the mean expression of target genes, but also the variance of their expression levels. Their model proposed a threshold effect of absolute gene expression on phenotype, where the availability of the transcripts dictated cell fate. Penetrance of the mutant trait was therefore determined by quantifiable variance in gene expression levels.

Variation, in genetic and phenotypic terms, has long been considered an important component of population fitness and adaptability. Similarly, one way to interpret the association between expression variance and phenotype is to consider how this might play a role in determining phenotype. Consider a pathway that plays a role in a developmental process or in a cell's response to a particular environmental stimulus. If the genes in that pathway have very low variance, a natural interpretation is that those genes are themselves highly constrained, and that the spectrum of potential responses from activation of that pathway is itself limited.

This interpretation was explored by examining the difference in gene expression variance in neural stem cells and fibroblasts derived from patients suffering from Schizophrenia (SZ), patients suffering from Parkinson's disease (PD), and healthy donors (controls). We demonstrate how gene expression variance can be used as a way to distinguish between phenotypic groups, the way in which constraint provides information about network topology, and to provide insight into the mechanisms associated with disease and normal states.

Of course it should be noted that any analysis of variance must be carefully considered in light of the methods used to collect and analyze biological datasets. Variation in biological systems has long been considered “noise” to be minimized either through careful experimental design or through the use of data normalization methods designed to improve comparisons between individual samples. However variation includes both biological and experimental (or random) effects and it is the former, rather than the latter which is important in the current study.

In our analysis, we considered highly-constrained and lowly-constrained genes, descriptors synonymous with low variance and high-variance states, respectively. These definitions, in part, helped to define our hypothesis: that the degree of variation in the expression of the genes associated with a particular cellular network is indicative of the plasticity [12] of that network. In this sense, high variance is associated with increased plasticity and low variance with diminished plasticity.

Our approach provides important evidence supporting the hypothesis that variation is an essential feature of biological systems and one that influences disease phenotype. In particular we illustrated that patterns of variance in certain key pathways are not random, but provide a potential mechanistic understanding of the phenotypic differences that arise in the development and progression of particular neurological diseases.

## Results

### Variance is not distributed randomly across signaling networks

The olfactory neuroepithelium is a continually regenerating tissue, and stem cells isolated from biopsied material give rise to neurons and glia in culture [13], and in transplant models in the rat [14], [15]. Patient-derived human olfactory neurosphere-derived (hONS) cells have been shown to be an informative tissue-specific system for studying the etiology of human brain disorders like PD and SZ [16], [17]. In a previous study, gene expression data from these hONS stem cell lines were used to identify disease-specific cellular alterations by comparing absolute expression profiles of hONS between donor groups [16]. Here, we use this same data set to focus instead on patterns of variability as a means to assess how hONS deviate from the normal population in PD and SZ donors, and explore the implications that variability may have on these disease processes.

Tissue biopsies from skin (fibroblasts) or the neuroepithelium of the nose were obtained from nine Schizophrenia (SZ) and eleven healthy control donors. Olfactory biopsies were taken from an additional thirteen donors with Parkinson's disease (PD) [16]. Adult stem cell lines are grown from olfactory biopsies for several passages as primary cultures, then moved through a neurosphere process to enrich for neural stem cells [13]. hONS cells are monolayer cultures expanded from disaggregated neurospheres [13], [16]. Patient-specific lines were grown from primary olfactory mucosa biopsies (primary) and hONS from all donor groups, additionally skin fibroblast cell lines were grown from the SZ patients and control donors. Genome-wide transcriptional profiling was performed on individual donor lines with replication (see Material and Methods). Principal component analysis (Figs. 4–6 in Text S1) shows that the samples clustered by the disease status of the donors. All donors used in this analysis were male.

**Figure 1:**
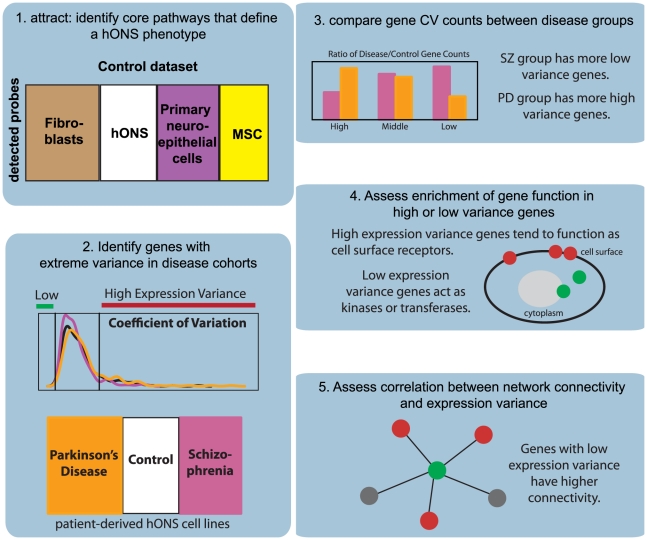
Overview of analysis pipeline.

**Figure 2 pgen-1002207-g002:**
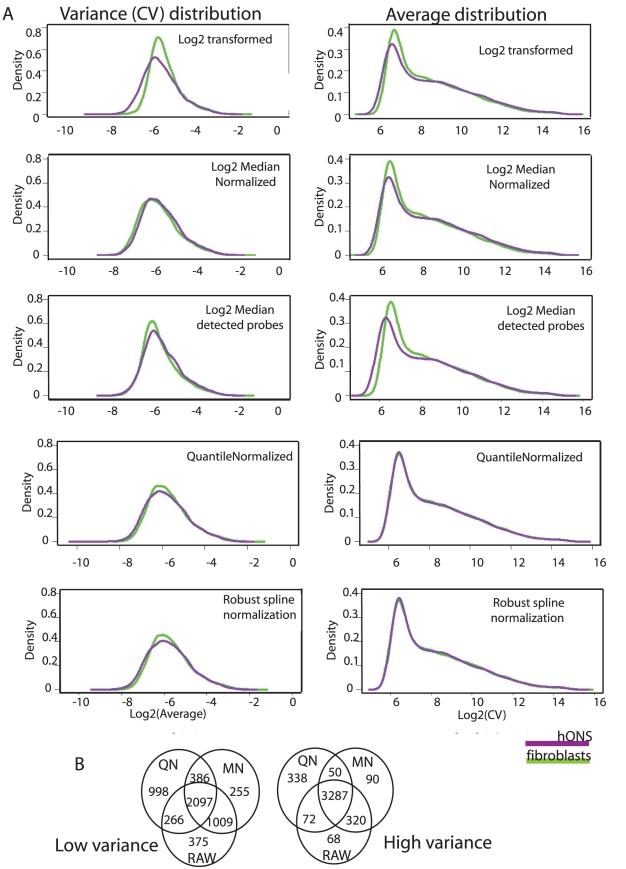
Expression variance is stable under different normalization strategies. A. Assessing the impact of normalization strategies on genome-wide variance distribution for the hONS stem cells and fibroblasts from the control group. B. Venn diagram demonstrating the geneset overlap of low-variance (left) or high-variance (right) identified from representative normalization strategies. There was a >75% concordance in the low-variance groups and a >90% concordance in the high-variance groups.

Figure 1 presents an outline of our analysis pipeline. We first examined the genome-wide expression variance distributions between skin fibroblasts and hONS derived from the same donors in the control group (Figure 2). Of the 22,184 probes represented on the Illumina microarray; 14,986 probes were detected in at least one cell type. To minimize experimental effects in our analysis, great care was taken to standardize our laboratory protocols and the assays that were performed. To explore the potential contributions of experimental noise, we consider a number of normalization approaches for microarray data and show that these effects do not contribute to the differences that we observe.

As a measure of variance, we used the coefficient of variation (CV) which is computed for each gene by dividing the standard deviation of its expression measures across a sample population by its average expression. We designate highly-constrained genes as those falling below the lower 25^th^ percentile of the genome-wide CV distribution based on all donors and lowly-constrained genes as those above the upper 25^th^ percentile; those genes in the range between the 25^th^ and 75^th^ percentile we refer to as the “Mid Variability” gene set. Basing our analysis on CV values protects against detecting patterns in variability influenced by trends in absolute expression alone (Text S1).

Normalization procedures are assumed to reduce or stabilize variance. To assess the impact of normalization on expression variance, CV distributions were examined using five normalization regimes and summarized in Figure 2: i) log2 transformed; ii) log2 transformed and median normalised in the presence or; iii) absence of non-detected probes; iv) log2 transformed and quantile normalized; v) log2 transformed and robust spline normalization. The distribution of expression values was consistent across all of the normalization strategies, which most likely reflects the high level of reproducibility of the raw data. CV was robust to the normalization strategy used but was most impacted by background correction or detection thresholds when using median normalization: this is discussed further in the Text S1, however all subsequent analyses were run on log2 transformed, quantile normalized data. Background subtraction was not performed, but data was thresholded using the Illumina detection scores. While the absolute numbers of genes in the ‘low’, ‘medium’ and ‘high’ variance categories varied slightly between the normalization methods, concordance was high (Figure 2B >75% overlap low-variance genes; >85% overlap high-variance genes) and the patterns in the underlying data types were highly reproducible (Figs. 1–3 in Text S1).

Using gene expression data from hONS and skin fibroblasts isolated from control donors, we next isolated the core attractor pathways whose differential expression distinguish a normal hONS stem cell expression phenotype, using the *attract* method that we recently developed [18]. Rather than testing individual genes, *attract* begins by using an ANOVA based method to test gene sets defined by KEGG pathways for their ability to distinguish between phenotypic states. Pathways ranked as significant are then each decomposed into “synexpression groups”—subsets with expression profiles that are both highly correlated and informative for distinguishing between phenotypes. These synexpression groups are then expanded to include genes with highly correlated profiles from within the original dataset, producing a collection of “core pathway modules.”

The top five *attract* modules were the MAPK signaling pathway, the focal adhesion pathway, the purine metabolism pathway, the neurotrophin signaling pathway and the cell cycle pathway (Table 1), each of which has previously been linked to important aspects of stem cell biology [19]–[21]. These modules were significantly different between the cell types tested at the 0.05 level (after adjusting for multiple testing using the Benjamini-Hochberg method). They were also the most “representative pathways,” in the sense that they contained the largest numbers of genes in the list of array probes significant between classes.

**Table 1:** The top 21 most discriminating KEGG pathways between the neuronal stem cells obtained from the disease and healthy control donor groups.

Rank	KEGG Pathway ID	KEGG Pathway Name	Adjusted p-value	Number of Detected Illumina IDs
1	4010	MAPK signaling pathway	0.01111	235
2	4510	Focal adhesion	0.03578	193
3	230	Purine metabolism	0.02908	143
4	4722	Neurotrophin signaling pathway	0.0003308	137
5	4110	Cell cycle	0.009490	132
6	4012	ErbB signaling pathway	0.001835	94
7	240	Pyrimidine metabolism	0.009490	88
8	4912	GnRH signaling pathway	0.04171	86
9	5220	Chronic myeloid leukemia	0.04171	86
10	5322	Systemic lupus erythematosus	0.0003308	76
11	4210	Apoptosis	0.04171	74
12	5221	Acute myeloid leukemia	0.04171	58
13	480	Glutathione metabolism	0.02949	45
14	3030	DNA replication	0.0003311	36
15	4672	Intestinal immune network for IgA production	0.01143	32
16	3440	Homologous recombination	0.002277	27
17	5332	Graft-versus-host disease	0.04447	27
18	5330	Allograft rejection	0.009490	26
19	5320	Autoimmune thyroid disease	0.03522	26
20	3430	Mismatch repair	0.009490	25
21	5310	Asthma	0.009490	15

All pathways were significant at the 0.05 level (after adjusting for multiple testing using the Benjamini-Hochberg method) and were ranked by the number of detected probes represented.

Overall we found 21 significant pathways (adjusted P-values <0.05). However, many of these pathways were overlapping in their gene content (see Table S1) and together represent three key common biological themes—immune response, growth factor signaling, and DNA replication—that are consistent with the phenotypic differences between the cell types.

**Figure 3:**
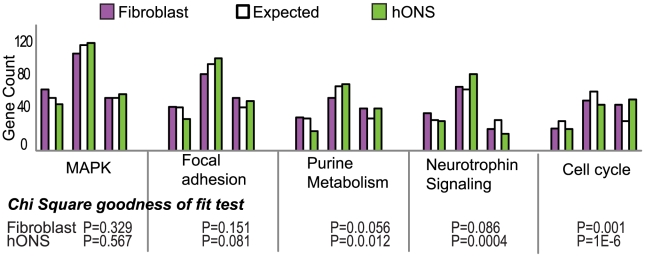
Distribution of variance patterns in the top 5 attract pathways for hONS and fibroblast cells. Chi-square goodness of fit was used to determine if variance profiles differed significantly from the expected 25∶50∶25% patterns.

In overlaying variance onto these networks we noted that the numbers of high-variance or highly constrained genes did not follow the expected population patterns, with a trend towards more high-variance genes across most of the pathways (Figure 3; Fig. 7 in Text S1). The purine metabolism and cell cycle pathways in particular had fewer low-variance and more high-variance genes than expected (Chi-square goodness of fit P-value <0.01 and P-value <1.2×10^−6^ respectively), an observation consistent in the hONS and skin fibroblast datasets. In contrast, the neurotrophin signaling pathway contained far fewer high-variance genes than expected (Chi-square goodness of fit P-value <0.01) suggesting that this network was under greater regulatory constraint.

Any analysis of variance must consider the contribution of technical variation to the data. To address this issue we compared the magnitude of the intra-individual expression variance with the size of the inter-individual expression variance associated with each donor group. Four additional donors had each contributed two independently derived biopsies, resulting in four replicate samples per donor (see Material and Methods and Text S3). The intra-individual mean of the CV distribution was smaller than the inter-individual CV mean, confirming that the technical variation in this data set was less than the biological variation observed. We next examined whether this observation held up when controlling for differences in sample size, by constructing variance distributions based on the same number of samples (*n* = 4), and found this trend persisted. Amongst the four individuals, differences in the CV distributions, albeit slight were observed for the five core pathway modules. Collectively these results suggest that repeated sampling of the same individual is associated with less expression variance, pointing to a strong genetic component in biological variability.

### Disease status alters the variability profiles in core pathway modules

**Figure 4:**
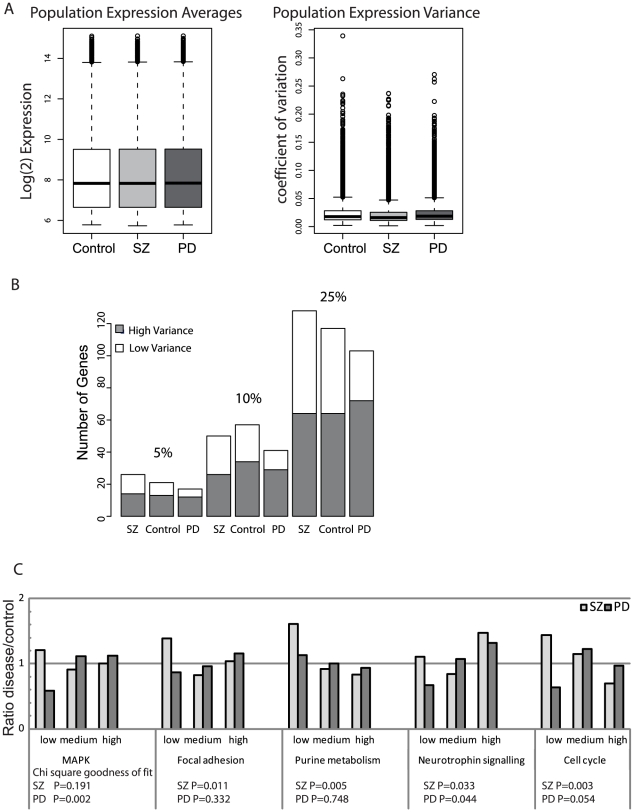
Expression variance discriminates between disease groups. A. Box plots showing whole-genome phenotype-specific mean expression levels and the corresponding CVs for hONS cells derived from the Control, PD, and SZ patient groups. As can be seen, there is no difference in the average log_2_(expression) measures but noticeable differences in the CVs. Using a two-sample t-test, there is a significant difference between the SZ and Control groups (p-value<2.2×10^−16^) and the PD and Control groups (p-value<0.0013). B. The observation of increase in high-variance genes for PD and increase in low-variance genes for SZ persists with different percentile cutoffs applied to the variance distribution in the MAPK signaling pathway. C. Ratios of gene counts for the attract pathways, showing a trend towards high-variance genes for PD and a significant increase in low-variance genes for SZ.

The distributions of expression variance for each of the patient groups highlighted an unexpected observation. Using a two sample t-test on log2 transformed CV values, we saw significant deviations from the control pattern in the SZ group (P-value <2.2×10^−16^), and also the PD group (P-value <0.001). This suggested that the SZ hONS lines demonstrated much less variation in their genome-wide expression patterns than was expected and in contrast, the PD hONS lines showed greater variation in their genome-wide expression patterns (Figure 4A; Text S2). Applying the same tests to identify disease-specific differences in genome-wide average expression showed no significant differences in either donor group.

We then investigated whether the differences in expression variance observed at a whole genome level were also apparent in our five core stem cell networks (Table 1). For each of these, we found that the SZ and PD groups sit at opposite ends of the variability spectrum (Figure 4C). The SZ group had a marked reduction in variance signaling pathway, as evidenced by more highly-constrained genes whereas the PD group had greater variance than the control group. The deviation in frequency distributions between the disease group and the control group was statistically significant for both SZ and PD groups in hONS stem cells, as assessed by a Chi-squared test with two degrees of freedom.

One might anticipate that increased expression variance, as seen in the PD group, is evidence of poor network integrity, a model that has been suggested as important in complex diseases. What was surprising, however, is the observation that the SZ group has significantly reduced expression variability in these core pathways, suggesting that both extremes of the variability spectrum may be implicated in disease processes.

These disease-specific patterns were independent on the particular cut-off used to define the regions of high and low constraint in the expression variance distribution (Figure 4). When more stringent cut-offs were applied (e.g. 5% and 10%) the same general trend of reduced variance for the SZ group and increased variance for the PD group was still observed.

### Highly-constrained and lowly-constrained genes have different functional roles as reflected by their position in cellular networks

Disease status was associated with different proportions of lowly-constrained and highly-constrained genes; this observation raised the possibility that these two classes may play distinct roles in maintaining or driving cellular phenotype. Using Gene Ontology terms (cellular component, molecular function and biological process categories) [22], we performed a representation analysis for each set of highly-constrained and lowly-constrained genes in the pathway modules for the three donor groups. We then mapped these genes to networks based on highly-annotated protein-protein interaction data and compared the patterns of transcriptional constraint between phenotypic states.

We found that genes with high-variance/low constraints were both functionally and physically involved at the periphery of signal transduction pathways. In hONS cells, they functioned largely as cell surface receptors and tended to be localized in the membrane, transmembrane or extracellular matrix regions (Table S2). This might suggest that the hONS were heterogeneous for expression of growth factor receptors and as a population had dynamic interactions with the extracellular environment. In contrast, highly-constrained genes tend to function in signaling roles, such as protein kinases and phosphatases (see Text S4). This might imply that all of the cells in the hONS population were competent to transduce signals through the MAPK pathway, and were only restricted by the expression of receptors or the availability of ligands.

The hONS stem cells derived from SZ donors demonstrated both loss of variability at the cell surface, and increased constraint in the intracellular signaling molecules. For MAPK signaling, we saw significant functional enrichment of signaling GO categories (Table S2) for low-variance genes in the SZ and control groups, while there was enrichment for high-variance genes in the PD group. This suggests that MAPK signaling is particularly important for distinguishing between these three groups.

**Figure 5:**
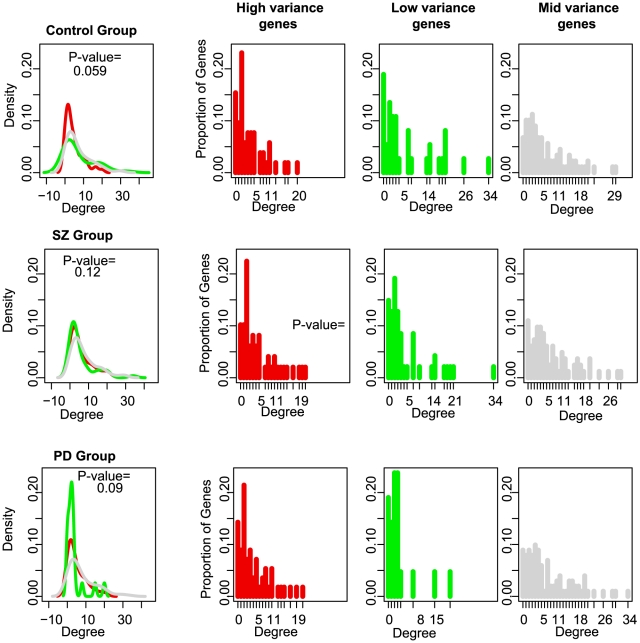
MAPK interaction networks and degree distribution density curves for the highly-constrained (red) and lowly-constrained (green) genes. p-values assess the significance of how different the two degree distributions are. The “Mid Variability” genes, falling between the 25th and 75th percentile of CV values, are shown in gray for comparison.

Just as cellular distribution of highly-constrained genes was not random, we observed a nonrandom pattern in the degree-distribution (connectivity) of genes based on their expression variance (Figure 5). The lowly-constrained genes had on average, a low degree whereas the highly-constrained genes were more highly connected to other genes in the network; this shift from a random distribution was marginally statistically significant for the control group (Chi-squared test; P-value  = 0.05958 for control group, P-value  = 0.1219 for SZ group, P-value  = 0.09595 for PD group, see Figs. 19 and 20 in Text S1), suggesting that not only are constraints imposed on genes linked to specific functional roles but they also have significantly distinct network topologies. Each disease group was associated with distinct deviations in degree distributions from those observed for the control group; the degree distributions between high and low-variance gene sets became more identical for the SZ group whereas in the PD group, the high and low-variance distributions appeared reversed from those observed in the control group. These observations suggest two different vehicles in which normal regulatory control through a signaling network may be disrupted or perturbed.

In comparing our three patient groups, we observed a consistent correlation between the degree of a particular gene and its expression variance, suggesting that transcriptional variability is an inherent aspect of all cellular systems (Fig. 20 in Text S1). This is consistent with our intuition that genes that are highly connected, and therefore play a central role in signaling or other networks, must be more tightly regulated than those that play more peripheral roles, including as cell surface receptors and downstream effectors.

It may be that the increased expressivity of those genes peripheral to the network provides a wider range of potential phenotypic response to external stimuli. In this way, disease processes that alter the variability in expression of particular genes may influence phenotype so that reduced expressivity limits the spectrum of response while increased expressivity tends toward loss of regulation over the cell's end-stage response.

## Discussion

In genetics, the concepts of expressivity and penetrance describe phenotype variability between individuals with shared genotypes and across populations, respectively. The implicit explanations for phenotypic variability are differences in genetic, epistatic, or epigenetic interactions. In genomic analysis, we often see this variability in terms of sequence or structural polymorphisms within the genome [23] and focus our efforts on understanding the link between genetic variation and phenotypic diversity. In this context, low genetic variation leads to poor evolutionary fitness, whereas the convergence of multiple variants on disease networks is increasingly thought to contribute to disease states [24], [25].

A key element in the central dogma of molecular biology is the role played by RNA as an intermediary between gene and protein (and ultimately with phenotype). In this light it is surprising that variability of expression levels has received so little attention. This may reflect the fact that in the analysis of gene expression data, variance is often associated with technical artifacts rather than being seen as an intrinsic property that reflects the normal range of phenotypic heterogeneity. If technical noise intrinsic to the platform was the major driver in variance, then one would expect these random effects to affect experimental samples equally. Indeed, as our goal was to do comparisons between phenotypic groups, we made every effort to minimize experimental noise, beginning with standardized protocols for sample collection and laboratory handling, quality control during RNA extraction, and labeling and hybridization together so as to avoid potential batch effects.

Given that we see specific biases in the variance profiles that correlate in a meaningful way with phenotype argues that this is not the case. Our results suggest that this variability is, in fact, a much more important measure of phenotype than previously recognized and that changes in the spectrum of expressivity may be fundamentally linked to the development of distinct phenotypic states.

The expressivity of individual genes such as the pluripotency factors Oct4 and Nanog has been highlighted by others as an important contributor to phenotypic robustness of a population of embryonic stem cells. Variance of gene expression in these cells is both predictable and essential – providing a dynamic range of pluripotency factors which is directly linked to the differentiation potential of individual cells within that population [26], [27]. If expression variance of one or two key regulators is an important modifier of phenotype, then it holds that this can be measured at a pathway level as well. Even across cells derived from different donors, the distribution of expression variance for a pathway is predictable, and deviations from this correlate with disease phenotypes. What are the likely sources of this ‘regulated noise’? The reduced variance observed between hONS lines derived from different biopsies from the same donor indicates a role for genetics. One possible scenario is that genetic polymorphism might impose a combinatorial impact on the expressivity of genes within a network, and the consequent alteration of dynamic range of that network outcome. Indeed, the degree of expression variance of a reporter construct was found to be a heritable trait in *S. cerevisiae* [28]. Epigenetic factors are also likely to play a key role in expressivity at individual loci, and others have shown stochastic, population-wide variance in epigenetic modification of key developmental loci. With increasing evidence that expression variance is an important phenotypic attribute of cell populations, it holds that variance profiles may also reflect abnormal genetic or epigenetic events contributing to disease phenotypes.

In this context, it is perhaps not surprising that the most profound shifts in expression variance were found in hONS cells isolated from SZ donors. SZ is a life-long psychotic disorder, with age of onset in males in early adulthood and later in females. SZ is considered a disease of neurodevelopment, based on epidemiological, histological and genetic evidence [29]–[32]. It is clear that SZ is a complex genetic disease with a strong environmental component. There are several well replicated genome-wide studies that have implicated common polymorphisms in the etiology of the disease, although these account for a small component of the heritability and an emerging theory is that polygenic risks explain more of the genetic component of this disease. Reduced expression variance of hONS networks provides fresh clues into potential mechanisms underlying diseases like SZ. For example, if the patterns of gene expressivity found in the hONS reflect those found in the developing brain, then the orderly cascade of brain development may be altered. In addition, it is feasible that an overly constrained biological pathway would be less adept at buffering environmental stressors.

Neuronal stem cells are the progenitors for neural cell states and the development of brain is rooted in the cell fate decisions that occur in these stem cells. Olfactory stem cells have been shown to serve as a good surrogate for neuronal stem cells and stem cell differentiation[14]. Our analysis of gene expression in olfactory stem cells cultured from patients with SZ, PD, and matched controls identified a number of key pathways that distinguished these three groups, including key signaling and developmental pathways. What was surprising about these pathways was that not only was there a notable difference in expression, but that expressivity, or variability in gene expression levels, was also significantly different between these different diseases. Further, when the expressivity was mapped to protein-protein interaction networks, there were distinctly different patterns of transcriptional constraint that depended on the connectivity of the proteins in the network. Further, these patterns depended not only on disease state, but also on the degree of connectivity within the corresponding protein-protein interaction pathway.

In examining the network topology, we found that in the SZ patients, there was significantly greater regulatory control over genes at the highly-connected core of the corresponding pathways. For example, in the MAPK pathway, SZ patients exhibited far less expressivity in the kinases that represent the core of the pathway. On the other hand, the PD patients had significantly fewer constrained genes mapping to the same core pathway regions than did controls. The increased variance associated with some proteins such as receptors, may indicate that these proteins have fluctuating turnover rates in the cell populations, which in turn will influence the capacity of cells to interact with their environment.

These data, together, paint a very interesting and compelling picture of the mechanism associated with disease. One of the defining characteristics of SZ is the disruption of normal cognitive processes and this is reflected in fewer neural synapses in parts of the brain. While it is no doubt a leap to interpret our results as having a direct link to thought processes in the disease, one could imagine that a high degree of constraint in key transcription factor networks that play a role in cellular differentiation and developmental processes. One could imagine that highly constrained transcriptional networks in neuronal stem cells could reduce the spectrum of cellular phenotypes that could be derived from those stem cells and reduce the plasticity resulting in the brain and altering its potential responses and thought patterns.

It is worth noting that among the pathways that demonstrate highly-constrained gene expression in our SZ patients are those involved in signaling in cancer. One consequence of increased constraints, and reduced plasticity, is defects in the network are more likely to be disastrous. In this kind of model, defects in cancer-related networks would lead to loss of those cells, preventing the types of adaption observed in cancer development. Although there are conflicting reports regarding the risk of cancer in schizophrenics, the majority of reports suggest that patients with SZ are protected against cancer in general, and from lung and colorectal, despite increased smoking [33], [34] and drinking habits [35] in this population. Laboratory studies have also reported reduced tumor growth in animal models of schizophrenia [36]. Although speculative, the high degree of transcriptional constraint we found in the MAPK pathway in SZ patients may explain, in part, these observations.

In contrast, disease states may arise where increased variance changes the predictability of network outcomes, resulting in dysregulation of the desired cell state. In PD, we observed an increase in the expression variance of core signaling pathways, which we predict will diminish the robustness of the network to external events. This may be an essential element that is shared amongst diseases of aging.

Although SZ and PD represent very different conditions, our results suggest that changes in expressivity relative to the normal spectrum of variability, may play an important role in the development of disease phenotypes. One possible way to interpret this is in the context of models first proposed by Conrad Waddington [37] and later refined by Stuart Kauffman [38]. Waddington and Kauffman envisioned what we can interpret as a gene expression state-space landscape defined by possible gene expression states. In this landscape, stable cell states represent fixed points (Kauffman referred to these as “attractors”) connected by evolutionarily-defined “canals” representing the differentiation pathways connecting distinct cell phenotypes. In this model, the SZ patients would be characterized by more highly-constrained, “deeper,” canals, limiting the potential end states that one might achieve during differentiation. PD patients, on the other hand, could be characterized by a flattening of the same canals (what has been referred to as “decanalization”), increasing dysregulation of the associated pathways and potentially allowing for a degradation of the well-defined cellular end states that might be available. While the potential importance of decanalization in disease has been discussed [25], we believe that our results are the first to suggest that over-canalization may be an equally important process in developing disease phenotypes.

## Materials and Methods

### Ethics statement

This work uses public expression datasets from patient-derived cells which were collected under the ethics approval of the Griffith University ethics committee.

### Gene expression data set

Illumina Human-Refseq8 v2 BeadChips (Illumina, Inc.) arrays were used to capture genome-wide gene expression profiles; the raw data was summarized using BeadStudio Version 3.1.7(Illumina, Inc). Background correction and normalization methods were performed using the R/Bioconductor lumi package. All downstream analyses were performed using Quantile normalized data, without background correction, and only probes passing the Illumina detection threshold were included in variance analysis. A probes was considered to pass the Illumina detection score if it had a detection p-value ≤0.01 in at least 75% of individuals in the same donor group, resulting in 14,986 probes. This expression data is available from ArrayExpress under the experiment accession number E-TABM-724.

### Intra-individual variance

Illumina Human-Refseq8 v3 BeadChips (Illumina, Inc.) arrays were used to collect genome-wide gene expression profiles of the four replicate samples for each of the four donors. The donors were made up of two healthy controls and two PD patients, and each donor underwent two independently derived biopsies that were each replicated twice, giving rise to four samples per donor. The raw gene expression data set was summarized as above. The data set was filtered using the same detection filter, and the subset of 6,809 detected probes common to the v3 and v2 arrays was used for the comparison of intra-individual and inter-individual variance analysis. To gauge the effect of sample size on variance, the inter-individual variance was calculated by computing CV distributions based on a random subset of 4 individuals from each donor group, and comparing these to the intra-individual CV distributions that were calculated from the four samples for each individual. From the 100 random subsets generated, we observed a reduction in the difference between the inter-individual variance and the intra-individual variance when the sample size was reduced and fixed at four replicates.

### Attract method

The attract package can be obtained from Bioconductor [http://www.bioconductor.org/packages/devel/bioc/html/attract.html] and is available as a module in the MeV microarray analysis software [39] (http://www.tm4.org/mev). To identify activated core pathways whose expression defines a control hONS phenotype, we ran attract on an expression data set consisting of the skin fibroblasts and two types of hONS lines from the control patients and a set of mesenchymal stem cell lines from a group of unrelated individuals. Attract was run using pathway modules defined by KEGG pathways represented in Bioconductor (version 2.4.1, Biobase version 2.8.0 and illuminaHumanv2BeadID.db version 1.6.0).

### Identifying low- and high-expression variance genes

A CV value was calculated for each detected probe by dividing the standard deviation of its expression in a donor group by its average group expression. Low and high-expression variance genes were identified as those genes below and above the 25^th^ percentile of the genome-wide CV distribution based on values from all donors.

### Assessing the significance of disease status versus healthy control group

P-values were obtained by comparing the counts of high, medium and low constraint genes in each of the control, SZ and PD groups and using a Chi-squared goodness of fit test where counts from the control group were designated as the expected counts.

### Representational analysis

For each of the top five pathway modules, we applied a representational analysis to the set of highly-constrained and lowly-constrained genes for each of the donor groups. We used tools from the Bioconductor GOstats package (version 2.14.0, run on R version 2.11.1). P-values were adjusted for using the Benjamini-Hochberg method within each ontology class, donor group and pathway module (Cellular Component, Molecular Function, or Biological Process) and significant results obtained at the 0.05 level. To focus on what functional terms were unique to genes of altered constraint, we excluded significant GO terms that appeared in both lists of highly-constrained and lowly-constrained genes and retained only those GO terms that were unique to each list. The significant GO terms for highly-constrained genes appear in the Supplementary File.

### Network topology p-values

A Kolmogorov-Smirnov test was used to test the degree distributions of the lowly and highly-constrained gene sets. A Gaussian kernel density estimator (the density function from R, using the default method to select bandwidth size) was used to produce the degree distribution density plots shown in Figure 4.

### Literature-curated networks

Protein-protein interactions were defined using two knowledge-based annotation systems, Ingenuity Pathway Analysis (IPA) software and the GeneGo metacore tool. Both tools permitted identification of highly curated protein-protein interactions; using IPA we extracted the degree of connectivity for each gene in the attract-networks and an image of the interaction network was obtained from GeneGo.

## Supporting Information

Table S1 Matrix of attract pathways with overlapping members and broad biological grouping.
(PDF)

Table S2 Functional enrichment (GO) tables.
(PDF)

Text S1 Normalization supplement: commentary on the impact of different normalization methodologies on variance distributions at a global and pathway level.
(PDF)

Text S2 Supplementary discussion on comparing gene counts between donor groups.
(PDF)

Text S3 Discussion of intra-donor variance patterns from additional donors with independently derived duplicate biopsies, and comparison to the between-donor variance patterns.
(PDF)

Text S4 Supplementary tutorial on calculating the degree distribution.
(PDF)
