# Supplementary material for: Variance of Gene Expression Identifies Altered Network Constraints in Neurological Disease
Source: PLoS Genet. 2011 Aug 11;7(8):e1002207. doi: 10.1371/journal.pgen.1002207 (PMC3154954; doi:10.1371/journal.pgen.1002207)
Supplement: Table S1 — Matrix of attract pathways with overlapping members and broad biological grouping. (PDF) [file pgen.1002207.s001.pdf]

		4722	5322	3030	4012	3440	4110	240	5330	3430	5310	4010	4672	230	480	5320	4510	4912	5220	4210	5221	5332
4722	Neurotrophin signaling pathway	137	0	0	50	0	11	0	0	0	0	64	0	0	0	0	45	41	43	21	30	0
5322	Systemic lupus erythematosus	0	76	0	0	0	0	0	16	0	13	1	15	0	0	15	2	0	0	1	0	14
3030	DNA replication	0	0	36	0	8	11	10	0	18	0	0	0	10	0	0	0	0	0	0	0	0
4012	ErbB signaling pathway	50	0	0	94	0	7	0	0	0	0	40	0	0	0	0	53	33	47	12	35	0
3440	Homologous recombination	0	0	8	0	27	0	4	0	8	0	0	0	4	0	0	0	0	0	0	0	0
4110	Cell cycle	11	0	11	7	0	132	0	0	3	0	6	0	0	0	0	4	0	23	0	3	0
240	Pyrimidine metabolism	0	0	10	0	4	0	88	0	4	0	0	0	64	3	0	0	0	0	0	0	0
5330	Allograft rejection	0	16	0	0	0	0	0	26	0	13	2	15	0	0	24	0	0	0	2	0	23
3430	Mismatch repair	0	0	18	0	8	3	4	0	25	0	0	0	4	0	0	0	0	0	0	0	0
5310	Asthma	0	13	0	0	0	0	0	13	0	15	1	12	0	0	12	0	0	0	1	0	11
4010	MAPK signaling pathway	64	1	0	40	0	6	0	2	0	1	235	1	0	0	1	48	48	34	24	23	4
4672	Intestinal immune network for IgA production	0	15	0	0	0	0	0	15	0	12	1	32	0	0	15	1	0	0	1	0	14
230	Purine metabolism	0	0	10	0	4	0	64	0	4	0	0	0	143	3	0	0	6	0	0	0	0
480	Glutathione metabolism	0	0	0	0	0	0	3	0	0	0	0	0	3	45	0	0	0	0	0	0	0
5320	Autoimmune thyroid disease	0	15	0	0	0	0	0	24	0	12	1	15	0	0	26	0	1	0	1	0	22
4510	Focal adhesion	45	2	0	53	0	4	0	0	0	0	48	1	0	0	0	193	26	33	15	25	0
4912	GnRH signaling pathway	41	0	0	33	0	0	0	0	0	0	48	0	6	0	1	26	86	14	2	14	0
5220	Chronic myeloid leukemia	43	0	0	47	0	23	0	0	0	0	34	0	0	0	0	33	14	86	18	37	0
4210	Apoptosis	21	1	0	12	0	0	0	2	0	1	24	1	0	0	1	15	2	18	74	15	4
5221	Acute myeloid leukemia	30	0	0	35	0	3	0	0	0	0	23	0	0	0	0	25	14	37	15	58	0
5332	Graft-versus-host disease	0	14	0	0	0	0	0	23	0	11	4	14	0	0	22	0	0	0	4	0	27

Immune Theme
DNA replication Theme
Growth factor Signaling Theme

30-50% overlap (max)
>= 50% (max)
