# Supplementary material for: Variance of Gene Expression Identifies Altered Network Constraints in Neurological Disease
Source: PLoS Genet. 2011 Aug 11;7(8):e1002207. doi: 10.1371/journal.pgen.1002207 (PMC3154954; doi:10.1371/journal.pgen.1002207)
Supplement: Table S2 — Functional enrichment (GO) tables. (PDF) [file pgen.1002207.s002.pdf]

Gene Ontology Enrichment Table – Control Low Variance Genes, Biological Process (BP)

GOID	AdjPval	Count	Size	Term
GO:0031657	0.001188	2	3	regulation of cyclin-dependent protein kinase activity during G1/S
GO:0031659	0.001188	2	3	positive regulation of cyclin-dependent protein kinase activity during G1/S
GO:0070141	0.001188	2	3	response to UV-A
GO:0045429	0.001359	3	17	positive regulation of nitric oxide biosynthetic process
GO:0008286	0.001364	4	45	insulin receptor signaling pathway
GO:0032868	0.001364	5	86	response to insulin stimulus
GO:0043122	0.001767	5	91	regulation of I-kappaB kinase/NF-kappaB cascade
GO:0032768	0.002421	3	21	regulation of monooxygenase activity
GO:0032663	0.002741	3	22	regulation of interleukin-2 production
GO:0045428	0.003056	3	23	regulation of nitric oxide biosynthetic process
GO:0045833	0.003056	3	23	negative regulation of lipid metabolic process
GO:0010827	0.003358	3	24	regulation of glucose transport
GO:0032623	0.003737	3	25	interleukin-2 production
GO:0009895	0.004138	3	26	negative regulation of catabolic process
GO:0007252	0.004599	2	6	I-kappaB phosphorylation
GO:0014002	0.004599	2	6	astrocyte development
GO:0045737	0.004599	2	6	positive regulation of cyclin-dependent protein kinase activity
GO:0032869	0.004599	4	65	cellular response to insulin stimulus
GO:0043434	0.004674	5	117	response to peptide hormone stimulus
GO:0051092	0.004849	3	28	positive regulation of NF-kappaB transcription factor activity
GO:0006809	0.005304	3	29	nitric oxide biosynthetic process
GO:0007249	0.005448	5	122	I-kappaB kinase/NF-kappaB cascade
GO:0046209	0.005695	3	30	nitric oxide metabolic process
GO:0051341	0.005695	3	30	regulation of oxidoreductase activity
GO:0071375	0.005695	4	70	cellular response to peptide hormone stimulus
GO:0042108	0.006561	3	32	positive regulation of cytokine biosynthetic process
GO:0010575	0.007067	2	8	positive regulation vascular endothelial growth factor production
GO:0014037	0.007067	2	8	Schwann cell differentiation
GO:0010573	0.008285	2	9	vascular endothelial growth factor production
GO:0010574	0.008285	2	9	regulation of vascular endothelial growth factor production
GO:0014072	0.008285	2	9	response to isoquinoline alkaloid
GO:0043278	0.008285	2	9	response to morphine
GO:0045086	0.008285	2	9	positive regulation of interleukin-2 biosynthetic process
GO:0050995	0.008285	2	9	negative regulation of lipid catabolic process
GO:0015758	0.00856	3	37	glucose transport
GO:0008645	0.009093	3	38	hexose transport

GO:0043123	0.009282	4	85	positive regulation of I-kappaB kinase/NF-kappaB cascade
GO:0015749	0.009585	3	39	monosaccharide transport
GO:0002711	0.009785	2	10	positive regulation of T cell mediated immunity
GO:0051291	0.010721	3	41	protein heterooligomerization
GO:0032770	0.011393	2	11	positive regulation of monooxygenase activity
GO:0042176	0.012467	3	44	regulation of protein catabolic process
GO:0051091	0.012467	3	44	positive regulation of transcription factor activity
GO:0090047	0.012467	3	44	positive regulation of transcription regulator activity
GO:0048708	0.012914	2	12	astrocyte differentiation
GO:0051051	0.012972	4	96	negative regulation of transport
GO:0045076	0.014672	2	13	regulation of interleukin-2 biosynthetic process
GO:0009411	0.014922	3	48	response to UV
GO:0042094	0.01599	2	14	interleukin-2 biosynthetic process
GO:0046627	0.01599	2	14	negative regulation of insulin receptor signaling pathway
GO:0050999	0.01599	2	14	regulation of nitric-oxide synthase activity
GO:0042035	0.01599	3	50	regulation of cytokine biosynthetic process
GO:0045765	0.01599	3	50	regulation of angiogenesis
GO:0043388	0.016688	3	51	positive regulation of DNA binding
GO:0002709	0.017579	2	15	regulation of T cell mediated immunity
GO:0010828	0.019156	2	16	positive regulation of glucose transport
GO:0045840	0.019156	2	16	positive regulation of mitosis
GO:0046326	0.019156	2	16	positive regulation of glucose import
GO:0046888	0.019156	2	16	negative regulation of hormone secretion
GO:0051785	0.019156	2	16	positive regulation of nuclear division
GO:0042089	0.020739	3	57	cytokine biosynthetic process
GO:0042107	0.020739	3	57	cytokine metabolic process
GO:0007346	0.020739	4	116	regulation of mitotic cell cycle
GO:0002824	0.020739	2	17	positive regulation of adaptive immune response based on somatic recombination of immune receptors built from immunoglobulin superfamily domains
GO:0046626	0.020739	2	17	regulation of insulin receptor signaling pathway
GO:0002821	0.022685	2	18	positive regulation of adaptive immune response
GO:0007613	0.022685	2	18	memory
GO:0051099	0.022872	3	60	positive regulation of binding
GO:0002260	0.024117	2	19	lymphocyte homeostasis
GO:0002705	0.024117	2	19	positive regulation of leukocyte mediated immunity
GO:0002708	0.024117	2	19	positive regulation of lymphocyte mediated immunity
GO:0051353	0.024117	2	19	positive regulation of oxidoreductase activity
GO:0008643	0.024117	3	62	carbohydrate transport
GO:0021782	0.024943	2	20	glial cell development
GO:0050994	0.024943	2	20	regulation of lipid catabolic process
GO:0008052	0.024943	1	1	sensory organ boundary specification
GO:0010160	0.024943	1	1	formation of organ boundary
GO:0014038	0.024943	1	1	regulation of Schwann cell differentiation

GO:0014040	0.024943	1	1	positive regulation of Schwann cell differentiation
GO:0021896	0.024943	1	1	forebrain astrocyte differentiation
GO:0021897	0.024943	1	1	forebrain astrocyte development
GO:0031999	0.024943	1	1	negative regulation of fatty acid beta-oxidation
GO:0033590	0.024943	1	1	response to cobalamin
GO:0043006	0.024943	1	1	activation of phospholipase A2 activity by calcium-mediated signaling
GO:0048859	0.024943	1	1	formation of anatomical boundary
GO:0060352	0.024943	1	1	cell adhesion molecule production
GO:0060353	0.024943	1	1	regulation of cell adhesion molecule production
GO:0060355	0.024943	1	1	positive regulation of cell adhesion molecule production
GO:0070162	0.024943	1	1	adiponectin secretion
GO:0070163	0.024943	1	1	regulation of adiponectin secretion
GO:0070164	0.024943	1	1	negative regulation of adiponectin secretion
GO:0070857	0.024943	1	1	regulation of bile acid biosynthetic process
GO:0070858	0.024943	1	1	negative regulation of bile acid biosynthetic process
GO:0002456	0.026164	2	21	T cell mediated immunity
GO:0045766	0.026164	2	21	positive regulation of angiogenesis
GO:0090068	0.026164	2	21	positive regulation of cell cycle process
GO:0046324	0.030161	2	23	regulation of glucose import
GO:0045862	0.031943	2	24	positive regulation of proteolysis
GO:0046323	0.031943	2	24	glucose import
GO:0050796	0.031943	2	24	regulation of insulin secretion
GO:0001776	0.033836	2	25	leukocyte homeostasis
GO:0045732	0.033836	2	25	positive regulation of protein catabolic process
GO:0090276	0.033836	2	25	regulation of peptide hormone secretion
GO:0002791	0.035739	2	26	regulation of peptide secretion
GO:0090087	0.035739	2	26	regulation of peptide transport
GO:0009894	0.036843	3	79	regulation of catabolic process
GO:0002699	0.037202	2	27	positive regulation of immune effector process
GO:0033619	0.039451	2	28	membrane protein proteolysis
GO:0043279	0.039451	2	28	response to alkaloid
GO:0002439	0.039715	1	2	chronic inflammatory response to antigenic stimulus
GO:0006924	0.039715	1	2	activation-induced cell death of T cells
GO:0006975	0.039715	1	2	DNA damage induced protein phosphorylation
GO:0007402	0.039715	1	2	ganglion mother cell fate determination
GO:0010470	0.039715	1	2	regulation of gastrulation
GO:0014050	0.039715	1	2	negative regulation of glutamate secretion
GO:0031558	0.039715	1	2	induction of apoptosis in response to chemical stimulus
GO:0032431	0.039715	1	2	activation of phospholipase A2 activity
GO:0034114	0.039715	1	2	regulation of heterotypic cell-cell adhesion
GO:0034116	0.039715	1	2	positive regulation of heterotypic cell-cell adhesion
GO:0046322	0.039715	1	2	negative regulation of fatty acid oxidation
GO:0060559	0.039715	1	2	positive regulation of calcidiol 1-monooxygenase activity

GO:0070586	0.039715	1	2	cell-cell adhesion involved in gastrulation
GO:0070587	0.039715	1	2	regulation of cell-cell adhesion involved in gastrulation
GO:0006941	0.039878	2	29	striated muscle contraction
GO:0002706	0.041877	2	30	regulation of lymphocyte mediated immunity
GO:0019915	0.041877	2	30	lipid storage
GO:0031331	0.048439	2	33	positive regulation of cellular catabolic process

Gene Ontology Enrichment Table – Control Low Variance Genes, Cellular Component (CC)

GOID	AdjPval	Count	Size	Term
GO:0008385	0.002487	2	4	IkappaB kinase complex

Gene Ontology Enrichment Table – Control Low Variance Genes, Molecule Function (MF)

GOID	AdjPval	Count	Size	Term
GO:0030235	0.001415	2	5	nitric-oxide synthase regulator activity
GO:0005149	0.002004	2	6	interleukin-1 receptor binding
GO:0004710	0.017124	1	1	MAP/ERK kinase kinase activity
GO:0008332	0.017124	1	1	low voltage-gated calcium channel activity
GO:0035255	0.017124	1	1	ionotropic glutamate receptor binding
GO:0005006	0.030019	1	2	epidermal growth factor receptor activity
GO:0008339	0.030019	1	2	MP kinase activity
GO:0008384	0.030019	1	2	IkappaB kinase activity
GO:0005070	0.040889	2	35	SH3/SH2 adaptor activity
GO:0031434	0.041517	1	3	mitogen-activated protein kinase kinase binding
GO:0035254	0.041517	1	3	glutamate receptor binding

Gene Ontology Enrichment Table – Control High Variance Genes, Biological Process (BP)

GOID	AdjPval	Count	Size	Term
GO:0018107	0.000140718	4	19	peptidyl-threonine phosphorylation
GO:0018210	0.000192973	4	21	peptidyl-threonine modification
GO:0002053	0.000759557	3	11	positive regulation of mesenchymal cell proliferation
GO:0070723	0.000776036	2	2	response to cholesterol
GO:0010463	0.000874825	3	12	mesenchymal cell proliferation
GO:0010464	0.000874825	3	12	regulation of mesenchymal cell proliferation
GO:0048010	0.001597448	3	15	vascular endothelial growth factor receptor signaling pathway
GO:0060389	0.001836573	3	16	pathway-restricted SMAD protein phosphorylation
GO:0043011	0.004765445	2	5	myeloid dendritic cell differentiation
GO:0001773	0.0065272	2	6	myeloid dendritic cell activation
GO:0060021	0.006852884	3	27	palate development
GO:0035265	0.008110066	3	29	organ growth
GO:0030031	0.009514431	4	70	cell projection assembly

GO:0007184	0.017602449	2	11	SMAD protein nuclear translocation
GO:0014855	0.017602449	2	11	striated muscle cell proliferation
GO:0055017	0.017602449	2	11	cardiac muscle tissue growth
GO:0060038	0.017602449	2	11	cardiac muscle cell proliferation
GO:0048705	0.017746338	4	87	skeletal system morphogenesis
GO:0060419	0.020179843	2	12	heart growth
GO:0002573	0.024393556	3	47	myeloid leukocyte differentiation
GO:0000188	0.032365529	2	16	inactivation of MAPK activity
GO:0048701	0.032365529	2	16	embryonic cranial skeleton morphogenesis
GO:0045778	0.03463407	2	17	positive regulation of ossification
GO:0060491	0.03463407	2	17	regulation of cell projection assembly
GO:0048008	0.036049042	2	18	platelet-derived growth factor receptor signaling pathway
GO:0048568	0.036049042	4	117	embryonic organ development
GO:0007257	0.036049042	2	19	activation of JUN kinase activity
GO:0002513	0.036049042	1	1	tolerance induction to self antigen
GO:0002649	0.036049042	1	1	regulation of tolerance induction to self antigen
GO:0002651	0.036049042	1	1	positive regulation of tolerance induction to self antigen
GO:0010693	0.036049042	1	1	negative regulation of alkaline phosphatase activity
GO:0031338	0.036049042	1	1	regulation of vesicle fusion
GO:0031340	0.036049042	1	1	positive regulation of vesicle fusion
GO:0042637	0.036049042	1	1	catagen
GO:0045602	0.036049042	1	1	negative regulation of endothelial cell differentiation
GO:0045743	0.036049042	1	1	positive regulation of fibroblast growth factor receptor signaling pathway
GO:0048818	0.036049042	1	1	positive regulation of hair follicle maturation
GO:0048819	0.036049042	1	1	regulation of hair follicle maturation
GO:0051794	0.036049042	1	1	regulation of catagen
GO:0051795	0.036049042	1	1	positive regulation of catagen
GO:0071425	0.036049042	1	1	hemopoietic stem cell proliferation
GO:0072074	0.036049042	1	1	kidney mesenchyme development
GO:0072075	0.036049042	1	1	metanephric mesenchyme development
GO:0072131	0.036049042	1	1	kidney mesenchyme morphogenesis
GO:0072132	0.036049042	1	1	mesenchyme morphogenesis
GO:0072133	0.036049042	1	1	metanephric mesenchyme morphogenesis
GO:0072185	0.036049042	1	1	metanephric cap development
GO:0072186	0.036049042	1	1	metanephric cap morphogenesis
GO:0090094	0.036049042	1	1	metanephric cap mesenchymal cell proliferation
GO:0090095	0.036049042	1	1	regulation of metanephric cap mesenchymal cell proliferation
GO:0090096	0.036049042	1	1	positive regulation of metanephric cap mesenchymal cell proliferation
GO:0046620	0.038470393	2	20	regulation of organ growth
GO:0048706	0.043630293	3	67	embryonic skeletal system development
GO:0030097	0.047367424	5	208	hemopoiesis
GO:0046632	0.048246459	2	24	alpha-beta T cell differentiation

Gene Ontology Enrichment Table – Control High Variance Genes, Cellular Component (CC)

GOID	AdjPval	Count	Size	Term
GO:0070022	0.004420915	2	3	transforming growth factor beta receptor complex

Gene Ontology Enrichment Table – Control High Variance Genes, Molecule Function (MF)

GOID	AdjPval	Count	Size	Term
GO:0005161	0.000135552	3	9	platelet-derived growth factor receptor binding
GO:0047498	0.000257261	2	2	calcium-dependent phospholipase A2 activity
GO:0005160	0.000298641	3	12	transforming growth factor beta receptor binding
GO:0005017	0.000587949	2	3	platelet-derived growth factor receptor activity
GO:0008330	0.000587949	2	3	protein tyrosine/threonine phosphatase activity
GO:0043121	0.000955089	2	4	neurotrophin binding
GO:0005021	0.001407019	2	5	vascular endothelial growth factor receptor activity
GO:0005114	0.00197638	2	6	type II transforming growth factor beta receptor binding
GO:0019992	0.00197638	2	6	diacylglycerol binding
GO:0046332	0.004422503	3	37	SMAD binding
GO:0048407	0.004866563	2	10	platelet-derived growth factor binding
GO:0050431	0.004866563	2	10	transforming growth factor beta binding
GO:0001847	0.014258425	1	1	opsonin receptor activity
GO:0004909	0.014258425	1	1	interleukin-1, Type I, activating receptor activity
GO:0005018	0.014258425	1	1	platelet-derived growth factor alpha-receptor activity
GO:0008331	0.014258425	1	1	high voltage-gated calcium channel activity
GO:0019955	0.017110184	3	68	cytokine binding
GO:0005019	0.025034523	1	2	platelet-derived growth factor beta-receptor activity
GO:0005030	0.025034523	1	2	neurotrophin receptor activity
GO:0005165	0.025034523	1	2	neurotrophin receptor binding
GO:0016019	0.025034523	1	2	peptidoglycan receptor activity
GO:0046582	0.025034523	1	2	Rap GTPase activator activity
GO:0070891	0.025034523	1	2	lipoteichoic acid binding
GO:0031625	0.034795646	2	34	ubiquitin protein ligase binding
GO:0004992	0.034795646	1	3	platelet activating factor receptor activity
GO:0005026	0.034795646	1	3	transforming growth factor beta receptor activity, type II
GO:0048406	0.034795646	1	3	nerve growth factor binding
GO:0004908	0.044036048	1	4	interleukin-1 receptor activity
GO:0051019	0.044036048	1	4	mitogen-activated protein kinase binding

Gene Ontology Enrichment Table – Parkinson's Disease Low Variance Genes, Biological Process (BP)

GOID	AdjPval	Count	Size	Term
GO:0002718	0.009392285	2	12	regulation of cytokine production during immune

				response
GO:0050810	0.010263949	2	13	regulation of steroid biosynthetic process
GO:0002367	0.012647612	2	15	cytokine production during immune response
GO:0045840	0.013774109	2	16	positive regulation of mitosis
GO:0051785	0.013774109	2	16	positive regulation of nuclear division
GO:0050715	0.01476835	2	17	positive regulation of cytokine secretion
GO:0002700	0.019060302	2	20	regulation of production of molecular mediator of immune response
GO:0019218	0.019955223	2	21	regulation of steroid metabolic process
GO:0050707	0.019955223	2	21	regulation of cytokine secretion
GO:0090068	0.019955223	2	21	positive regulation of cell cycle process
GO:0032663	0.021160392	2	22	regulation of interleukin-2 production
GO:0045833	0.022430066	2	23	negative regulation of lipid metabolic process
GO:0046324	0.022430066	2	23	regulation of glucose import
GO:0010827	0.023190904	2	24	regulation of glucose transport
GO:0046323	0.023190904	2	24	glucose import
GO:0032623	0.024513632	2	25	interleukin-2 production
GO:0002037	0.024653644	1	1	negative regulation of L-glutamate transport
GO:0008052	0.024653644	1	1	sensory organ boundary specification
GO:0010160	0.024653644	1	1	formation of organ boundary
GO:0021896	0.024653644	1	1	forebrain astrocyte differentiation
GO:0021897	0.024653644	1	1	forebrain astrocyte development
GO:0045994	0.024653644	1	1	positive regulation of translational initiation by iron
GO:0046136	0.024653644	1	1	positive regulation of vitamin metabolic process
GO:0048859	0.024653644	1	1	formation of anatomical boundary
GO:0060557	0.024653644	1	1	positive regulation of vitamin D biosynthetic process
GO:0070857	0.024653644	1	1	regulation of bile acid biosynthetic process
GO:0070858	0.024653644	1	1	negative regulation of bile acid biosynthetic process
GO:0050663	0.026026344	2	28	cytokine secretion
GO:0042108	0.032413032	2	32	positive regulation of cytokine biosynthetic process
GO:0015758	0.035901171	2	37	glucose transport
GO:0008645	0.035901171	2	38	hexose transport
GO:0002036	0.035901171	1	2	regulation of L-glutamate transport
GO:0002374	0.035901171	1	2	cytokine secretion during immune response
GO:0002439	0.035901171	1	2	chronic inflammatory response to antigenic stimulus
GO:0002739	0.035901171	1	2	regulation of cytokine secretion during immune response
GO:0002740	0.035901171	1	2	negative regulation of cytokine secretion during immune response
GO:0006447	0.035901171	1	2	regulation of translational initiation by iron
GO:0007402	0.035901171	1	2	ganglion mother cell fate determination
GO:0010470	0.035901171	1	2	regulation of gastrulation
GO:0030656	0.035901171	1	2	regulation of vitamin metabolic process
GO:0034114	0.035901171	1	2	regulation of heterotypic cell-cell adhesion
GO:0034116	0.035901171	1	2	positive regulation of heterotypic cell-cell adhesion

GO:0044070	0.035901171	1	2	regulation of anion transport
GO:0051956	0.035901171	1	2	negative regulation of amino acid transport
GO:0060544	0.035901171	1	2	regulation of necroptosis
GO:0060545	0.035901171	1	2	positive regulation of necroptosis
GO:0060553	0.035901171	1	2	induction of necroptosis
GO:0060555	0.035901171	1	2	induction of necroptosis by extracellular signals
GO:0060556	0.035901171	1	2	regulation of vitamin D biosynthetic process
GO:0060559	0.035901171	1	2	positive regulation of calcidiol 1-monooxygenase activity
GO:0070266	0.035901171	1	2	necroptosis
GO:0070586	0.035901171	1	2	cell-cell adhesion involved in gastrulation
GO:0070587	0.035901171	1	2	regulation of cell-cell adhesion involved in gastrulation
GO:0015749	0.036452944	2	39	monosaccharide transport
GO:0002440	0.037222005	2	40	production of molecular mediator of immune response
GO:0007088	0.041446693	2	43	regulation of mitosis
GO:0051783	0.041446693	2	43	regulation of nuclear division
GO:0002726	0.045914959	1	3	positive regulation of T cell cytokine production
GO:0006927	0.045914959	1	3	transformed cell apoptosis
GO:0010940	0.045914959	1	3	positive regulation of necrotic cell death
GO:0022011	0.045914959	1	3	myelination in the peripheral nervous system
GO:0032292	0.045914959	1	3	ensheathment of axons in the peripheral nervous system
GO:0034113	0.045914959	1	3	heterotypic cell-cell adhesion
GO:0042368	0.045914959	1	3	vitamin D biosynthetic process
GO:0045948	0.045914959	1	3	positive regulation of translational initiation
GO:0048853	0.045914959	1	3	forebrain morphogenesis
GO:0042035	0.048832966	2	50	regulation of cytokine biosynthetic process

Gene Ontology Enrichment Table – Parkinson’s Disease Low Variance Genes, Cellular Component (CC)

No significant results at the 0.05 P-value cut-off.

Gene Ontology Enrichment Table – Parkinson’s Disease Low Variance Genes, Molecule Function (MF)

GOID	AdjPval	Count	Size	Term
GO:0008349	0.014987477	1	1	MAP kinase kinase kinase kinase activity
GO:0005105	0.026089088	1	2	type 1 fibroblast growth factor receptor binding
GO:0005111	0.026089088	1	2	type 2 fibroblast growth factor receptor binding

Gene Ontology Enrichment Table – Parkinson’s Disease High Variance Genes, Biological Process (BP)

GOID	AdjPval	Count	Size	Term
GO:0007568	3.19E-06	8	85	aging

GO:0007184	1.37E-05	4	11	SMAD protein nuclear translocation
GO:0060389	6.05E-05	4	16	pathway-restricted SMAD protein phosphorylation
GO:0045740	9.47E-05	4	18	positive regulation of DNA replication
GO:0050678	0.000116754	5	40	regulation of epithelial cell proliferation
GO:0050679	0.000236855	4	23	positive regulation of epithelial cell proliferation
GO:0050673	0.000239743	5	47	epithelial cell proliferation
GO:0051272	0.000240189	6	79	positive regulation of cellular component movement
GO:0060021	0.000409903	4	27	palate development
GO:0032496	0.000496448	5	56	response to lipopolysaccharide
GO:0035265	0.00051925	4	29	organ growth
GO:0070723	0.00051925	2	2	response to cholesterol
GO:0014855	0.000553889	3	11	striated muscle cell proliferation
GO:0055017	0.000553889	3	11	cardiac muscle tissue growth
GO:0060038	0.000553889	3	11	cardiac muscle cell proliferation
GO:0030307	0.000566775	4	30	positive regulation of cell growth
GO:0040008	0.000669798	9	251	regulation of growth
GO:0060419	0.000700432	3	12	heart growth
GO:0045793	0.000703788	4	32	positive regulation of cell size
GO:0001558	0.000756131	7	147	regulation of cell growth
GO:0002237	0.000759985	5	63	response to molecule of bacterial origin
GO:0030335	0.00114841	5	70	positive regulation of cell migration
GO:0046777	0.00114841	5	70	protein amino acid autophosphorylation
GO:0051054	0.001215733	4	38	positive regulation of DNA metabolic process
GO:0048010	0.001215733	3	15	vascular endothelial growth factor receptor signaling pathway
GO:0022601	0.001215733	2	3	menstrual cycle phase
GO:0060045	0.001215733	2	3	positive regulation of cardiac muscle cell proliferation
GO:0048701	0.001416103	3	16	embryonic cranial skeleton morphogenesis
GO:0040017	0.001464486	5	75	positive regulation of locomotion
GO:0032570	0.001674512	3	17	response to progesterone stimulus
GO:0045778	0.001674512	3	17	positive regulation of ossification
GO:0007435	0.002184345	2	4	salivary gland morphogenesis
GO:0018107	0.002269684	3	19	peptidyl-threonine phosphorylation
GO:0006275	0.002269684	4	46	regulation of DNA replication
GO:0046620	0.002586341	3	20	regulation of organ growth
GO:0048705	0.002661878	5	87	skeletal system morphogenesis
GO:0018210	0.002873865	3	21	peptidyl-threonine modification
GO:0007431	0.003211868	2	5	salivary gland development
GO:0043011	0.003211868	2	5	myeloid dendritic cell differentiation
GO:0060390	0.003211868	2	5	regulation of SMAD protein nuclear translocation
GO:0060391	0.003211868	2	5	positive regulation of SMAD protein nuclear translocation
GO:0010720	0.003234973	4	52	positive regulation of cell development
GO:0000302	0.003624176	4	54	response to reactive oxygen species

GO:0045927	0.003624176	4	54	positive regulation of growth
GO:0001773	0.004408325	2	6	myeloid dendritic cell activation
GO:0033160	0.004408325	2	6	positive regulation of protein import into nucleus, translocation
GO:0055021	0.004408325	2	6	regulation of cardiac muscle tissue growth
GO:0055024	0.004408325	2	6	regulation of cardiac muscle tissue development
GO:0060043	0.004408325	2	6	regulation of cardiac muscle cell proliferation
GO:0060420	0.004408325	2	6	regulation of heart growth
GO:0003006	0.004408325	6	152	reproductive developmental process
GO:0048565	0.004754427	3	26	gut development
GO:0006935	0.005600417	5	107	chemotaxis
GO:0042330	0.005600417	5	107	taxis
GO:0010718	0.005698164	2	7	positive regulation of epithelial to mesenchymal transition
GO:0010770	0.005698164	2	7	positive regulation of cell morphogenesis involved in differentiation
GO:0033158	0.005698164	2	7	regulation of protein import into nucleus, translocation
GO:0051489	0.005698164	2	7	regulation of filopodium assembly
GO:0051491	0.005698164	2	7	positive regulation of filopodium assembly
GO:0051602	0.005698164	2	7	response to electrical stimulus
GO:0034097	0.006716018	4	66	response to cytokine stimulus
GO:0048706	0.007050699	4	67	embryonic skeletal system development
GO:0010092	0.007146768	2	8	specification of organ identity
GO:0010717	0.007146768	2	8	regulation of epithelial to mesenchymal transition
GO:0035272	0.007146768	2	8	exocrine system development
GO:0007565	0.00759613	4	69	female pregnancy
GO:0048568	0.00759613	5	117	embryonic organ development
GO:0048638	0.007687566	3	32	regulation of developmental growth
GO:0060395	0.008917412	2	9	SMAD protein signal transduction
GO:0042476	0.010501738	3	36	odontogenesis
GO:0001759	0.010712477	2	10	induction of an organ
GO:0010862	0.010712477	2	10	positive regulation of pathway-restricted SMAD protein phosphorylation
GO:0042542	0.011788551	3	38	response to hydrogen peroxide
GO:0014070	0.01198952	4	80	response to organic cyclic substance
GO:0002053	0.012310673	2	11	positive regulation of mesenchymal cell proliferation
GO:0007628	0.012310673	2	11	adult walking behavior
GO:0022612	0.012310673	2	11	gland morphogenesis
GO:0051052	0.0131405	4	83	regulation of DNA metabolic process
GO:0048738	0.013836396	3	41	cardiac muscle tissue development
GO:0010463	0.014106997	2	12	mesenchymal cell proliferation
GO:0010464	0.014106997	2	12	regulation of mesenchymal cell proliferation
GO:0060393	0.014106997	2	12	regulation of pathway-restricted SMAD protein phosphorylation
GO:0022602	0.014594491	3	42	ovulation cycle process

GO:0048511	0.015051886	4	87	rhythmic process
GO:0048562	0.015588411	4	88	embryonic organ morphogenesis
GO:0030501	0.015899114	2	13	positive regulation of bone mineralization
GO:0033993	0.015899114	2	13	response to lipid
GO:0048536	0.015899114	2	13	spleen development
GO:0070169	0.015899114	2	13	positive regulation of biomineral formation
GO:0042698	0.017854115	3	46	ovulation cycle
GO:0031128	0.017978658	2	14	developmental induction
GO:0045168	0.017978658	2	14	cell-cell signaling involved in cell fate specification
GO:0048286	0.020120691	2	15	lung alveolus development
GO:0070887	0.020186776	6	222	cellular response to chemical stimulus
GO:0048704	0.0204409	3	49	embryonic skeletal system morphogenesis
GO:0042692	0.022256467	4	100	muscle cell differentiation
GO:0000188	0.022256467	2	16	inactivation of MAPK activity
GO:0030035	0.024631135	2	17	microspike assembly
GO:0046847	0.024631135	2	17	filopodium assembly
GO:0034330	0.025286043	3	55	cell junction organization
GO:0002513	0.025286043	1	1	tolerance induction to self antigen
GO:0002649	0.025286043	1	1	regulation of tolerance induction to self antigen
GO:0002651	0.025286043	1	1	positive regulation of tolerance induction to self antigen
GO:0010693	0.025286043	1	1	negative regulation of alkaline phosphatase activity
GO:0019227	0.025286043	1	1	neuronal action potential propagation
GO:0031338	0.025286043	1	1	regulation of vesicle fusion
GO:0031340	0.025286043	1	1	positive regulation of vesicle fusion
GO:0035026	0.025286043	1	1	leading edge cell differentiation
GO:0042637	0.025286043	1	1	catagen
GO:0043932	0.025286043	1	1	ossification involved in bone remodeling
GO:0045602	0.025286043	1	1	negative regulation of endothelial cell differentiation
GO:0045743	0.025286043	1	1	positive regulation of fibroblast growth factor receptor signaling pathway
GO:0048818	0.025286043	1	1	positive regulation of hair follicle maturation
GO:0048819	0.025286043	1	1	regulation of hair follicle maturation
GO:0051365	0.025286043	1	1	cellular response to potassium ion starvation
GO:0051764	0.025286043	1	1	actin crosslink formation
GO:0051794	0.025286043	1	1	regulation of catagen
GO:0051795	0.025286043	1	1	positive regulation of catagen
GO:0060363	0.025286043	1	1	cranial suture morphogenesis
GO:0060364	0.025286043	1	1	frontal suture morphogenesis
GO:0070483	0.025286043	1	1	detection of hypoxia
GO:0071425	0.025286043	1	1	hemopoietic stem cell proliferation
GO:0048008	0.025286043	2	18	platelet-derived growth factor receptor signaling pathway
GO:0014706	0.025286043	4	107	striated muscle tissue development
GO:0022604	0.026021224	4	108	regulation of cell morphogenesis

GO:0030278	0.026021224	3	56	regulation of ossification
GO:0001837	0.033117812	2	21	epithelial to mesenchymal transition
GO:0010769	0.033365897	3	62	regulation of cell morphogenesis involved in differentiation
GO:0017156	0.035327537	2	22	calcium ion-dependent exocytosis
GO:0030500	0.035327537	2	22	regulation of bone mineralization
GO:0070167	0.035327537	2	22	regulation of biomineral formation
GO:0045216	0.04114707	2	24	cell-cell junction organization
GO:0046632	0.04114707	2	24	alpha-beta T cell differentiation
GO:0006979	0.04294357	4	128	response to oxidative stress
GO:0000189	0.04294357	1	2	nuclear translocation of MAPK
GO:0001661	0.04294357	1	2	conditioned taste aversion
GO:0001865	0.04294357	1	2	NK T cell differentiation
GO:0002266	0.04294357	1	2	follicular dendritic cell activation
GO:0002268	0.04294357	1	2	follicular dendritic cell differentiation
GO:0002514	0.04294357	1	2	B cell tolerance induction
GO:0002661	0.04294357	1	2	regulation of B cell tolerance induction
GO:0002663	0.04294357	1	2	positive regulation of B cell tolerance induction
GO:0007195	0.04294357	1	2	inhibition of adenylate cyclase activity by dopamine receptor signaling pathway
GO:0007403	0.04294357	1	2	glial cell fate determination
GO:0010692	0.04294357	1	2	regulation of alkaline phosphatase activity
GO:0014051	0.04294357	1	2	gamma-aminobutyric acid secretion
GO:0031392	0.04294357	1	2	regulation of prostaglandin biosynthetic process
GO:0031394	0.04294357	1	2	positive regulation of prostaglandin biosynthetic process
GO:0031620	0.04294357	1	2	regulation of fever
GO:0031622	0.04294357	1	2	positive regulation of fever
GO:0031650	0.04294357	1	2	regulation of heat generation
GO:0031652	0.04294357	1	2	positive regulation of heat generation
GO:0032792	0.04294357	1	2	negative regulation of CREB transcription factor activity
GO:0034616	0.04294357	1	2	response to laminar fluid shear stress
GO:0045113	0.04294357	1	2	regulation of integrin biosynthetic process
GO:0045726	0.04294357	1	2	positive regulation of integrin biosynthetic process
GO:0051136	0.04294357	1	2	regulation of NK T cell differentiation
GO:0051138	0.04294357	1	2	positive regulation of NK T cell differentiation
GO:0060083	0.04294357	1	2	smooth muscle contraction involved in micturition
GO:0045137	0.045815124	3	73	development of primary sexual characteristics

Gene Ontology Enrichment Table – Parkinson’s Disease High Variance Genes, Cellular Component (CC)

GOID	AdjPval	Count	Size	Term
GO:0070022	0.008406	2	3	transforming growth factor beta receptor complex

Gene Ontology Enrichment Table – Parkinson's Disease High Variance Genes, Molecule Function (MF)

GOID	AdjPval	Count	Size	Term
GO:0005160	9.65E-06	4	12	transforming growth factor beta receptor binding
GO:0005114	4.84E-05	3	6	type II transforming growth factor beta receptor binding
GO:0005161	0.000180884	3	9	platelet-derived growth factor receptor binding
GO:0050431	0.000234458	3	10	transforming growth factor beta binding
GO:0005165	0.000281281	2	2	neurotrophin receptor binding
GO:0046983	0.000319363	11	387	protein dimerization activity
GO:0019955	0.000522737	5	68	cytokine binding
GO:0046332	0.00056791	4	37	SMAD binding
GO:0005017	0.000690772	2	3	platelet-derived growth factor receptor activity
GO:0046982	0.001209262	6	130	protein heterodimerization activity
GO:0004908	0.001263181	2	4	interleukin-1 receptor activity
GO:0043121	0.001263181	2	4	neurotrophin binding
GO:0019900	0.001658041	6	141	kinase binding
GO:0005021	0.001901963	2	5	vascular endothelial growth factor receptor activity
GO:0034713	0.001901963	2	5	type I transforming growth factor beta receptor binding
GO:0005539	0.002296757	5	100	glycosaminoglycan binding
GO:0019992	0.002599688	2	6	diacylglycerol binding
GO:0070412	0.003477718	2	7	R-SMAD binding
GO:0001871	0.003477718	5	112	pattern binding
GO:0030247	0.003477718	5	112	polysaccharide binding
GO:0019901	0.0041654	5	117	protein kinase binding
GO:0019966	0.004366181	2	8	interleukin-1 binding
GO:0048407	0.006747977	2	10	platelet-derived growth factor binding
GO:0004675	0.010892995	2	13	transmembrane receptor protein serine/threonine kinase activity
GO:0005024	0.010892995	2	13	transforming growth factor beta receptor activity
GO:0019899	0.018698384	8	412	enzyme binding
GO:0001847	0.018698384	1	1	opsonin receptor activity
GO:0004909	0.018698384	1	1	interleukin-1, Type I, activating receptor activity
GO:0004910	0.018698384	1	1	interleukin-1, Type II, blocking receptor activity
GO:0005018	0.018698384	1	1	platelet-derived growth factor alpha-receptor activity
GO:0005163	0.018698384	1	1	nerve growth factor receptor binding
GO:0034987	0.018698384	1	1	immunoglobulin receptor binding
GO:0034988	0.018698384	1	1	Fc-gamma receptor I complex binding
GO:0034989	0.018698384	1	1	GTP-Ral binding
GO:0005019	0.032923541	1	2	platelet-derived growth factor beta-receptor activity
GO:0005030	0.032923541	1	2	neurotrophin receptor activity
GO:0016019	0.032923541	1	2	peptidoglycan receptor activity
GO:0046582	0.032923541	1	2	Rap GTPase activator activity

GO:0047498	0.032923541	1	2	calcium-dependent phospholipase A2 activity
GO:0070891	0.032923541	1	2	lipoteichoic acid binding
GO:0008201	0.033250987	3	74	heparin binding
GO:0030246	0.033789082	5	211	carbohydrate binding
GO:0004992	0.044045182	1	3	platelet activating factor receptor activity
GO:0005026	0.044045182	1	3	transforming growth factor beta receptor activity, type II
GO:0005078	0.044045182	1	3	MAP-kinase scaffold activity
GO:0008330	0.044045182	1	3	protein tyrosine/threonine phosphatase activity
GO:0017160	0.044045182	1	3	Ral GTPase binding
GO:0048406	0.044045182	1	3	nerve growth factor binding
GO:0004896	0.04525731	2	33	cytokine receptor activity

Gene Ontology Enrichment Table – Schizophrenia Low Variance Genes, Biological Process (BP)

GOID	AdjPval	Count	Size	Term
GO:0051291	0.001608	4	41	protein heterooligomerization
GO:0032663	0.003909	3	22	regulation of interleukin-2 production
GO:0045833	0.004331	3	23	negative regulation of lipid metabolic process
GO:0010827	0.004686	3	24	regulation of glucose transport
GO:0032623	0.005165	3	25	interleukin-2 production
GO:0007252	0.005872	2	6	I-kappaB phosphorylation
GO:0014002	0.005872	2	6	astrocyte development
GO:0010575	0.009666	2	8	positive regulation vascular endothelial growth factor production
GO:0010573	0.011767	2	9	vascular endothelial growth factor production
GO:0010574	0.011767	2	9	regulation of vascular endothelial growth factor production
GO:0014072	0.011767	2	9	response to isoquinoline alkaloid
GO:0043278	0.011767	2	9	response to morphine
GO:0045086	0.011767	2	9	positive regulation of interleukin-2 biosynthetic process
GO:0015758	0.013147	3	37	glucose transport
GO:0008645	0.013869	3	38	hexose transport
GO:0002711	0.013869	2	10	positive regulation of T cell mediated immunity
GO:0050996	0.013869	2	10	positive regulation of lipid catabolic process
GO:0015749	0.014627	3	39	monosaccharide transport
GO:0048708	0.018234	2	12	astrocyte differentiation
GO:0045076	0.02036	2	13	regulation of interleukin-2 biosynthetic process
GO:0051055	0.02036	2	13	negative regulation of lipid biosynthetic process
GO:0042094	0.023129	2	14	interleukin-2 biosynthetic process
GO:0002709	0.026143	2	15	regulation of T cell mediated immunity
GO:0010828	0.027461	2	16	positive regulation of glucose transport
GO:0045840	0.027461	2	16	positive regulation of mitosis
GO:0046326	0.027461	2	16	positive regulation of glucose import

GO:0046888	0.027461	2	16	negative regulation of hormone secretion
GO:0051785	0.027461	2	16	positive regulation of nuclear division
GO:0002824	0.030117	2	17	positive regulation of adaptive immune response based on somatic recombination of immune receptors built from immunoglobulin superfamily domains
GO:0045429	0.030117	2	17	positive regulation of nitric oxide biosynthetic process
GO:0002821	0.032936	2	18	positive regulation of adaptive immune response
GO:0007613	0.032936	2	18	memory
GO:0007346	0.033043	4	116	regulation of mitotic cell cycle
GO:0002705	0.033043	2	19	positive regulation of leukocyte mediated immunity
GO:0002708	0.033043	2	19	positive regulation of lymphocyte mediated immunity
GO:0008052	0.033043	1	1	sensory organ boundary specification
GO:0009814	0.033043	1	1	defense response, incompatible interaction
GO:0009816	0.033043	1	1	defense response to bacterium, incompatible interaction
GO:0010160	0.033043	1	1	formation of organ boundary
GO:0010511	0.033043	1	1	regulation of phosphatidylinositol biosynthetic process
GO:0010512	0.033043	1	1	negative regulation of phosphatidylinositol biosynthetic process
GO:0021896	0.033043	1	1	forebrain astrocyte differentiation
GO:0021897	0.033043	1	1	forebrain astrocyte development
GO:0043006	0.033043	1	1	activation of phospholipase A2 activity by calcium-mediated signaling
GO:0048859	0.033043	1	1	formation of anatomical boundary
GO:0060352	0.033043	1	1	cell adhesion molecule production
GO:0060353	0.033043	1	1	regulation of cell adhesion molecule production
GO:0060355	0.033043	1	1	positive regulation of cell adhesion molecule production
GO:0070162	0.033043	1	1	adiponectin secretion
GO:0070163	0.033043	1	1	regulation of adiponectin secretion
GO:0070164	0.033043	1	1	negative regulation of adiponectin secretion
GO:0070857	0.033043	1	1	regulation of bile acid biosynthetic process
GO:0070858	0.033043	1	1	negative regulation of bile acid biosynthetic process
GO:0008643	0.034875	3	62	carbohydrate transport
GO:0021782	0.034875	2	20	glial cell development
GO:0050994	0.034875	2	20	regulation of lipid catabolic process
GO:0051259	0.035404	4	122	protein oligomerization
GO:0002456	0.037164	2	21	T cell mediated immunity
GO:0032768	0.037164	2	21	regulation of monooxygenase activity
GO:0090068	0.037164	2	21	positive regulation of cell cycle process
GO:0045428	0.042222	2	23	regulation of nitric oxide biosynthetic process
GO:0046324	0.042222	2	23	regulation of glucose import
GO:0046323	0.045022	2	24	glucose import
GO:0050796	0.045022	2	24	regulation of insulin secretion
GO:0090276	0.04785	2	25	regulation of peptide hormone secretion

Gene Ontology Enrichment Table – Schizophrenia Low Variance Genes, Cellular Component (CC)

GOID	AdjPval	Count	Size	Term
GO:0008385	0.003902184	2	4	IkappaB kinase complex

Gene Ontology Enrichment Table – Schizophrenia Low Variance Genes, Molecule Function (MF)

GOID	AdjPval	Count	Size	Term
GO:0019209	3.89E-05	4	18	kinase activator activity
GO:0004708	0.000251915	3	11	MAP kinase kinase activity
GO:0004712	0.00038724	3	13	protein serine/threonine/tyrosine kinase activity
GO:0005078	0.000615237	2	3	MAP-kinase scaffold activity
GO:0005149	0.00248109	2	6	interleukin-1 receptor binding
GO:0019207	0.003398482	4	70	kinase regulator activity
GO:0019894	0.004061897	2	8	kinesin binding
GO:0004722	0.007897792	3	43	protein serine/threonine phosphatase activity
GO:0030295	0.010838366	2	14	protein kinase activator activity
GO:0030159	0.012034827	2	15	receptor signaling complex scaffold activity
GO:0004710	0.018364997	1	1	MAP/ERK kinase kinase activity
GO:0008332	0.018364997	1	1	low voltage-gated calcium channel activity
GO:0035255	0.018364997	1	1	ionotropic glutamate receptor binding
GO:0032947	0.019795345	2	21	protein complex scaffold
GO:0004705	0.030949549	1	2	JUN kinase activity
GO:0005006	0.030949549	1	2	epidermal growth factor receptor activity
GO:0008384	0.030949549	1	2	IkappaB kinase activity
GO:0015270	0.043821211	1	3	dihydropyridine-sensitive calcium channel activity
GO:0031434	0.043821211	1	3	mitogen-activated protein kinase kinase binding
GO:0035254	0.043821211	1	3	glutamate receptor binding

Gene Ontology Enrichment Table – Schizophrenia High Variance Genes, Biological Process (BP)

GOID	AdjPval	Count	Size	Term
GO:0007184	1.42E-05	4	11	SMAD protein nuclear translocation
GO:0000060	2.03E-05	5	28	protein import into nucleus, translocation
GO:0060389	5.26E-05	4	16	pathway-restricted SMAD protein phosphorylation
GO:0000302	0.000347241	5	54	response to reactive oxygen species
GO:0060021	0.000355903	4	27	palate development
GO:0070723	0.000526444	2	2	response to cholesterol
GO:0002053	0.000526444	3	11	positive regulation of mesenchymal cell proliferation
GO:0014855	0.000526444	3	11	striated muscle cell proliferation
GO:0055017	0.000526444	3	11	cardiac muscle tissue growth
GO:0060038	0.000526444	3	11	cardiac muscle cell proliferation
GO:0010463	0.00064043	3	12	mesenchymal cell proliferation

GO:0010464	0.00064043	3	12	regulation of mesenchymal cell proliferation
GO:0060419	0.00064043	3	12	heart growth
GO:0042542	0.00107459	4	38	response to hydrogen peroxide
GO:0048010	0.00116963	3	15	vascular endothelial growth factor receptor signaling pathway
GO:0016572	0.001244579	2	3	histone phosphorylation
GO:0022601	0.001244579	2	3	menstrual cycle phase
GO:0060045	0.001244579	2	3	positive regulation of cardiac muscle cell proliferation
GO:0048701	0.001342658	3	16	embryonic cranial skeleton morphogenesis
GO:0032570	0.0015368	3	17	response to progesterone stimulus
GO:0045778	0.0015368	3	17	positive regulation of ossification
GO:0060491	0.0015368	3	17	regulation of cell projection assembly
GO:0018107	0.002050005	3	19	peptidyl-threonine phosphorylation
GO:0002573	0.002050005	4	47	myeloid leukocyte differentiation
GO:0018210	0.002590007	3	21	peptidyl-threonine modification
GO:0030949	0.003085229	2	5	positive regulation of vascular endothelial growth factor receptor signaling pathway
GO:0043011	0.003085229	2	5	myeloid dendritic cell differentiation
GO:0060390	0.003085229	2	5	regulation of SMAD protein nuclear translocation
GO:0060391	0.003085229	2	5	positive regulation of SMAD protein nuclear translocation
GO:0003006	0.003288871	6	152	reproductive developmental process
GO:0001773	0.00429697	2	6	myeloid dendritic cell activation
GO:0030947	0.00429697	2	6	regulation of vascular endothelial growth factor receptor signaling pathway
GO:0033160	0.00429697	2	6	positive regulation of protein import into nucleus, translocation
GO:0055021	0.00429697	2	6	regulation of cardiac muscle tissue growth
GO:0055024	0.00429697	2	6	regulation of cardiac muscle tissue development
GO:0060043	0.00429697	2	6	regulation of cardiac muscle cell proliferation
GO:0060420	0.00429697	2	6	regulation of heart growth
GO:0048565	0.00429697	3	26	gut development
GO:0035265	0.005575927	3	29	organ growth
GO:0010718	0.005575927	2	7	positive regulation of epithelial to mesenchymal transition
GO:0010770	0.005575927	2	7	positive regulation of cell morphogenesis involved in differentiation
GO:0033158	0.005575927	2	7	regulation of protein import into nucleus, translocation
GO:0051489	0.005575927	2	7	regulation of filopodium assembly
GO:0051491	0.005575927	2	7	positive regulation of filopodium assembly
GO:0034097	0.005671382	4	66	response to cytokine stimulus
GO:0048706	0.005918788	4	67	embryonic skeletal system development
GO:0048568	0.006124229	5	117	embryonic organ development
GO:0051591	0.006561878	3	31	response to cAMP

GO:0010717	0.006944623	2	8	regulation of epithelial to mesenchymal transition
GO:0060395	0.008442885	2	9	SMAD protein signal transduction
GO:0042476	0.009065803	3	36	odontogenesis
GO:0031346	0.009586173	3	37	positive regulation of cell projection organization
GO:0010862	0.009886088	2	10	positive regulation of pathway-restricted SMAD protein phosphorylation
GO:0030097	0.011771083	6	208	hemopoiesis
GO:0048738	0.011960364	3	41	cardiac muscle tissue development
GO:0022602	0.012497205	3	42	ovulation cycle process
GO:0048562	0.012497205	4	88	embryonic organ morphogenesis
GO:0060393	0.012808397	2	12	regulation of pathway-restricted SMAD protein phosphorylation
GO:0030501	0.014812746	2	13	positive regulation of bone mineralization
GO:0070169	0.014812746	2	13	positive regulation of biomineral formation
GO:0048704	0.017705377	3	49	embryonic skeletal system morphogenesis
GO:0030099	0.020346522	4	104	myeloid cell differentiation
GO:0010720	0.020346522	3	52	positive regulation of cell development
GO:0000188	0.020515152	2	16	inactivation of MAPK activity
GO:0048754	0.022202653	3	54	branching morphogenesis of a tube
GO:0030035	0.02251371	2	17	microspike assembly
GO:0046847	0.02251371	2	17	filopodium assembly
GO:0060349	0.02251371	2	17	bone morphogenesis
GO:0034330	0.022737669	3	55	cell junction organization
GO:0030278	0.023769775	3	56	regulation of ossification
GO:0001763	0.024244663	3	58	morphogenesis of a branching structure
GO:0002513	0.024244663	1	1	tolerance induction to self antigen
GO:0002649	0.024244663	1	1	regulation of tolerance induction to self antigen
GO:0002651	0.024244663	1	1	positive regulation of tolerance induction to self antigen
GO:0010693	0.024244663	1	1	negative regulation of alkaline phosphatase activity
GO:0031338	0.024244663	1	1	regulation of vesicle fusion
GO:0031340	0.024244663	1	1	positive regulation of vesicle fusion
GO:0035026	0.024244663	1	1	leading edge cell differentiation
GO:0042637	0.024244663	1	1	catagen
GO:0043932	0.024244663	1	1	ossification involved in bone remodeling
GO:0045602	0.024244663	1	1	negative regulation of endothelial cell differentiation
GO:0045743	0.024244663	1	1	positive regulation of fibroblast growth factor receptor signaling pathway
GO:0048818	0.024244663	1	1	positive regulation of hair follicle maturation
GO:0048819	0.024244663	1	1	regulation of hair follicle maturation
GO:0051365	0.024244663	1	1	cellular response to potassium ion starvation
GO:0051764	0.024244663	1	1	actin crosslink formation
GO:0051794	0.024244663	1	1	regulation of catagen
GO:0051795	0.024244663	1	1	positive regulation of catagen

GO:0060363	0.024244663	1	1	cranial suture morphogenesis
GO:0060364	0.024244663	1	1	frontal suture morphogenesis
GO:0070483	0.024244663	1	1	detection of hypoxia
GO:0071425	0.024244663	1	1	hemopoietic stem cell proliferation
GO:0072074	0.024244663	1	1	kidney mesenchyme development
GO:0072075	0.024244663	1	1	metanephric mesenchyme development
GO:0072131	0.024244663	1	1	kidney mesenchyme morphogenesis
GO:0072132	0.024244663	1	1	mesenchyme morphogenesis
GO:0072133	0.024244663	1	1	metanephric mesenchyme morphogenesis
GO:0072185	0.024244663	1	1	metanephric cap development
GO:0072186	0.024244663	1	1	metanephric cap morphogenesis
GO:0090094	0.024244663	1	1	metanephric cap mesenchymal cell proliferation
GO:0090095	0.024244663	1	1	regulation of metanephric cap mesenchymal cell proliferation
GO:0090096	0.024244663	1	1	positive regulation of metanephric cap mesenchymal cell proliferation
GO:0046620	0.026715557	2	20	regulation of organ growth
GO:0002521	0.02707879	4	119	leukocyte differentiation
GO:0001837	0.028945749	2	21	epithelial to mesenchymal transition
GO:0002062	0.030748571	2	22	chondrocyte differentiation
GO:0030500	0.030748571	2	22	regulation of bone mineralization
GO:0070167	0.030748571	2	22	regulation of biomineral formation
GO:0045216	0.035712031	2	24	cell-cell junction organization
GO:0046632	0.035712031	2	24	alpha-beta T cell differentiation
GO:0045137	0.039446867	3	73	development of primary sexual characteristics
GO:0001658	0.040059862	2	26	branching involved in ureteric bud morphogenesis
GO:0060675	0.040059862	2	26	ureteric bud morphogenesis
GO:0031344	0.040059862	3	74	regulation of cell projection organization
GO:0000189	0.040059862	1	2	nuclear translocation of MAPK
GO:0001661	0.040059862	1	2	conditioned taste aversion
GO:0001865	0.040059862	1	2	NK T cell differentiation
GO:0002266	0.040059862	1	2	follicular dendritic cell activation
GO:0002268	0.040059862	1	2	follicular dendritic cell differentiation
GO:0002514	0.040059862	1	2	B cell tolerance induction
GO:0002661	0.040059862	1	2	regulation of B cell tolerance induction
GO:0002663	0.040059862	1	2	positive regulation of B cell tolerance induction
GO:0006975	0.040059862	1	2	DNA damage induced protein phosphorylation
GO:0007195	0.040059862	1	2	inhibition of adenylate cyclase activity by dopamine receptor signaling pathway
GO:0007403	0.040059862	1	2	glial cell fate determination
GO:0010692	0.040059862	1	2	regulation of alkaline phosphatase activity
GO:0031392	0.040059862	1	2	regulation of prostaglandin biosynthetic process
GO:0031394	0.040059862	1	2	positive regulation of prostaglandin biosynthetic process
GO:0031620	0.040059862	1	2	regulation of fever

GO:0031622	0.040059862	1	2	positive regulation of fever
GO:0031650	0.040059862	1	2	regulation of heat generation
GO:0031652	0.040059862	1	2	positive regulation of heat generation
GO:0032792	0.040059862	1	2	negative regulation of CREB transcription factor activity
GO:0034616	0.040059862	1	2	response to laminar fluid shear stress
GO:0045113	0.040059862	1	2	regulation of integrin biosynthetic process
GO:0045726	0.040059862	1	2	positive regulation of integrin biosynthetic process
GO:0051136	0.040059862	1	2	regulation of NK T cell differentiation
GO:0051138	0.040059862	1	2	positive regulation of NK T cell differentiation
GO:0090100	0.045690848	2	29	positive regulation of transmembrane receptor protein serine/threonine kinase signaling pathway
GO:0001558	0.046052212	4	147	regulation of cell growth
GO:0030307	0.047909693	2	30	positive regulation of cell growth

Gene Ontology Enrichment Table – Schizophrenia High Variance Genes, Cellular Component (CC)

GOID	AdjPval	Count	Size	Term
GO:0070022	0.012637	2	3	transforming growth factor beta receptor complex

Gene Ontology Enrichment Table – Schizophrenia High Variance Genes, Molecule Function (MF)

GOID	AdjPval	Count	Size	Term
GO:0017017	8.89E-10	6	10	MAP kinase tyrosine/serine/threonine phosphatase activity
GO:0033549	8.89E-10	6	10	MAP kinase phosphatase activity
GO:0004725	9.05E-07	8	80	protein tyrosine phosphatase activity
GO:0008138	9.86E-07	6	32	protein tyrosine/serine/threonine phosphatase activity
GO:0005160	9.80E-06	4	12	transforming growth factor beta receptor binding
GO:0005114	4.31E-05	3	6	type II transforming growth factor beta receptor binding
GO:0050431	0.00018446	3	10	transforming growth factor beta binding
GO:0046332	0.000463453	4	37	SMAD binding
GO:0008330	0.000670493	2	3	protein tyrosine/threonine phosphatase activity
GO:0043121	0.001134359	2	4	neurotrophin binding
GO:0034713	0.001784542	2	5	type I transforming growth factor beta receptor binding
GO:0070412	0.003387225	2	7	R-SMAD binding
GO:0001847	0.016901677	1	1	opsonin receptor activity
GO:0004909	0.016901677	1	1	interleukin-1, Type I, activating receptor activity
GO:0005018	0.016901677	1	1	platelet-derived growth factor alpha-receptor activity
GO:0034987	0.016901677	1	1	immunoglobulin receptor binding

GO:0034988	0.016901677	1	1	Fc-gamma receptor I complex binding
GO:0034989	0.016901677	1	1	GTP-Ral binding
GO:0005030	0.029179947	1	2	neurotrophin receptor activity
GO:0005165	0.029179947	1	2	neurotrophin receptor binding
GO:0016019	0.029179947	1	2	peptidoglycan receptor activity
GO:0046582	0.029179947	1	2	Rap GTPase activator activity
GO:0047498	0.029179947	1	2	calcium-dependent phospholipase A2 activity
GO:0070891	0.029179947	1	2	lipoteichoic acid binding
GO:0005017	0.040694835	1	3	platelet-derived growth factor receptor activity
GO:0005026	0.040694835	1	3	transforming growth factor beta receptor activity, type II
GO:0017160	0.040694835	1	3	Ral GTPase binding
GO:0048406	0.040694835	1	3	nerve growth factor binding
