# Supplementary material for: Variance of Gene Expression Identifies Altered Network Constraints in Neurological Disease
Source: PLoS Genet. 2011 Aug 11;7(8):e1002207. doi: 10.1371/journal.pgen.1002207 (PMC3154954; doi:10.1371/journal.pgen.1002207)
Supplement: Text S1 — Normalization supplement: commentary on the impact of different normalization methodologies on variance distributions at a global and pathway level. (PDF) [file pgen.1002207.s003.pdf]

Normalization of microarray data typically consists of a series of data transformations intended to aid in the comparison of gene expression data gathered across a series of hybridizations. These may include background correction to remove geographical biases in fluorescent intensity, applying intensity thresholds or flooring to remove poorly detected probes and to increase signal-noise sensitivity, log transformation to normalize the distribution of probes across the intensity range of the experiment, and the scaling of data such that information extracted from one array slide is equivalent to that extracted from another in the series. There are several standard approaches to array normalization which are generally accepted in the scaling of expression intensities, but the impact of these transformations on expression variance is less well understood. A reasonable assumption is that the process of thresholding, scaling and log transforming data will reduce variance between samples.

We examined the impact of each of these data transformation steps on the variance structure of hONS datasets. Each sample includes technical replicates which are averaged for the purposes of summarizing the outcomes of each normalization step. Groups are collapsed into disease parameter. Note the high reproducibility of the raw data across the entire dataset.

Figure 1: Raw data, log2 transformed, no background correction. CV (left), average intensity (right).

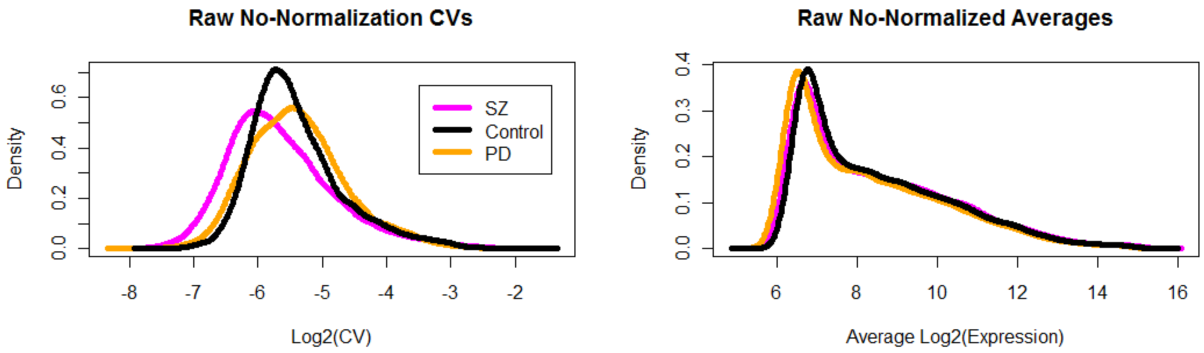

Figure 2: Background subtraction using the R/Lumi normalization package. CV (left), average intensity (right). The data structure is strikingly different with bimodal distribution of CV and of average intensities.

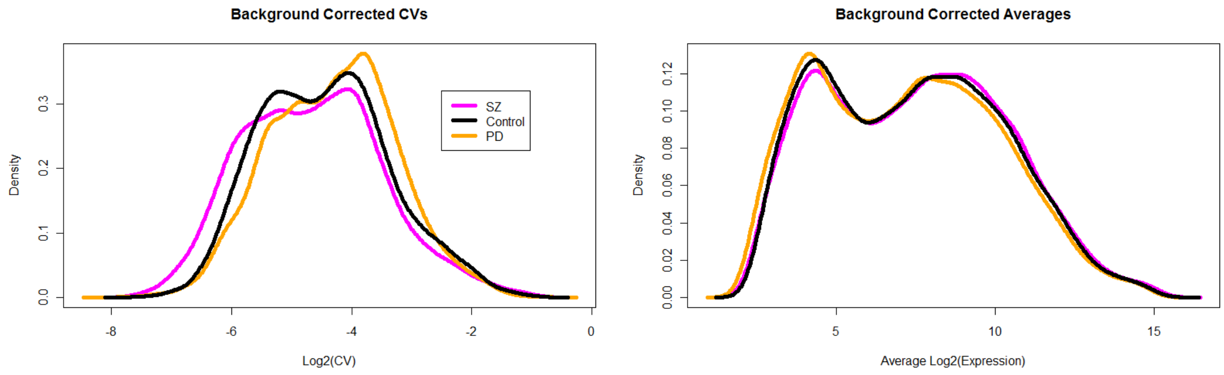

Background correction is a hang-over from spotted arrays, which were susceptible to very large variations in signal intensity across the slide. The next-generation of bead arrays are not processed in an equivalent manner, and detection of a probe is determined by a signal intensity above background probes, as well as the reproducibility of hybridization across the large number of probes represented on the bead array. Both background subtraction and thresholding of probes based on a detection p-value had a large impact on variance. Background correction was applied using the (bgAdjust.affy in lumi).

The log2-median transformation is the ssn (simple scaling normalization) method in lumi. It takes the non-logged expression value and divides it by the ratio of its column (sample) median to the mean of all the sample medians. The log2 transform that's applied afterwards means that you're taking log2(expression) – log2(ratio).

Figure 3A and 3B: The impact of Background subtraction on median-scaled data. CV (left), average intensity (right). Top panels background corrected data, bottom panels tranformation applied to the detected probes only, no background correction.

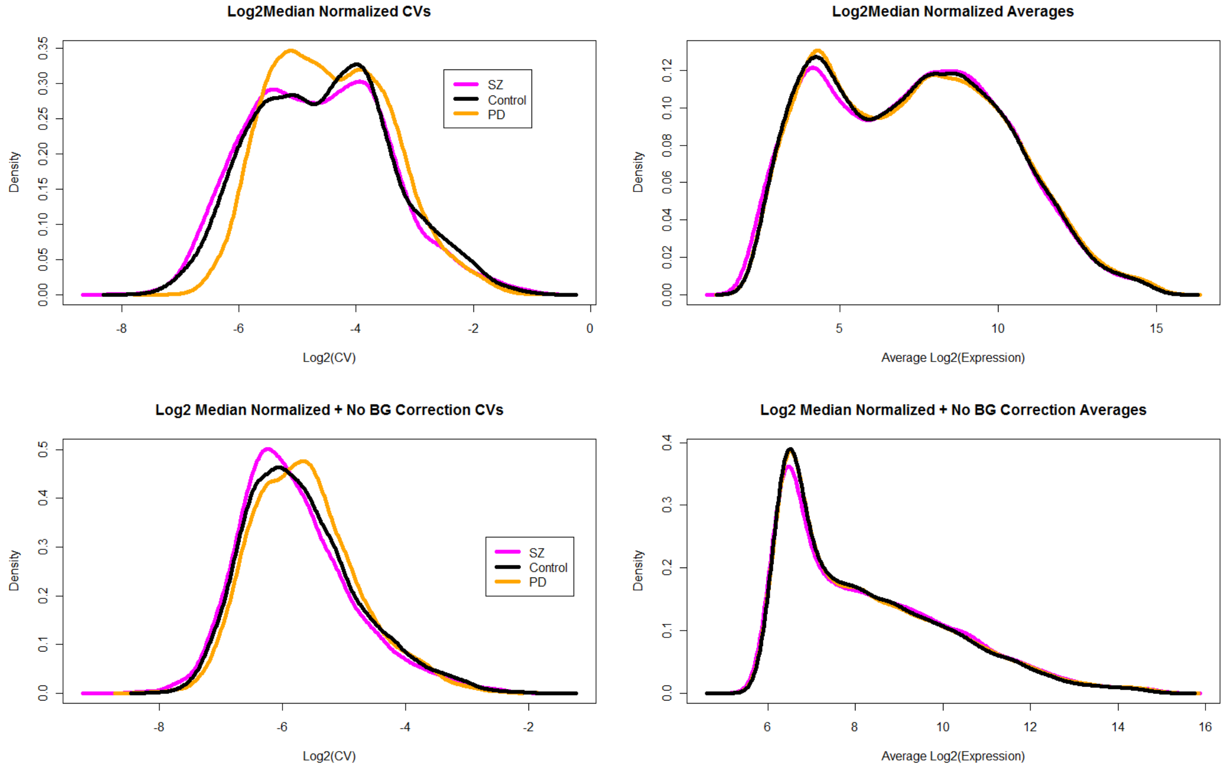

Quantile normalization, and variations on this such as robust spline normalization, take into account intensity-dependant differences that might be required for data scaling.

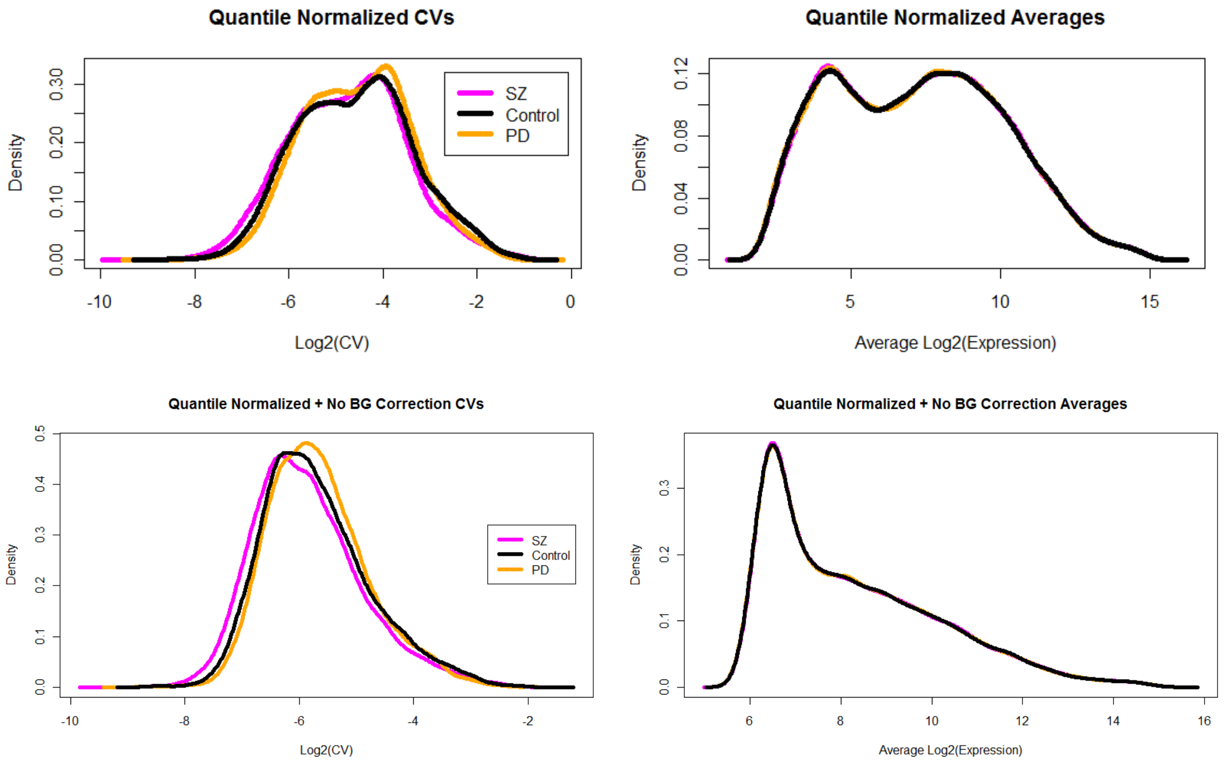

The impact of these data transformations is to reduce global differences between the samples. This is illustrated in a series of principle component analyses, demonstrating the increasing compactness of the samples from Raw data (panel 1) to quantile normalized (panel 6). In each normalization group, the background-subtracted data is the lower panel.

PCA on Raw (No-Normalization) Data Set, Tech Reps Averaged + Detected Probes Only

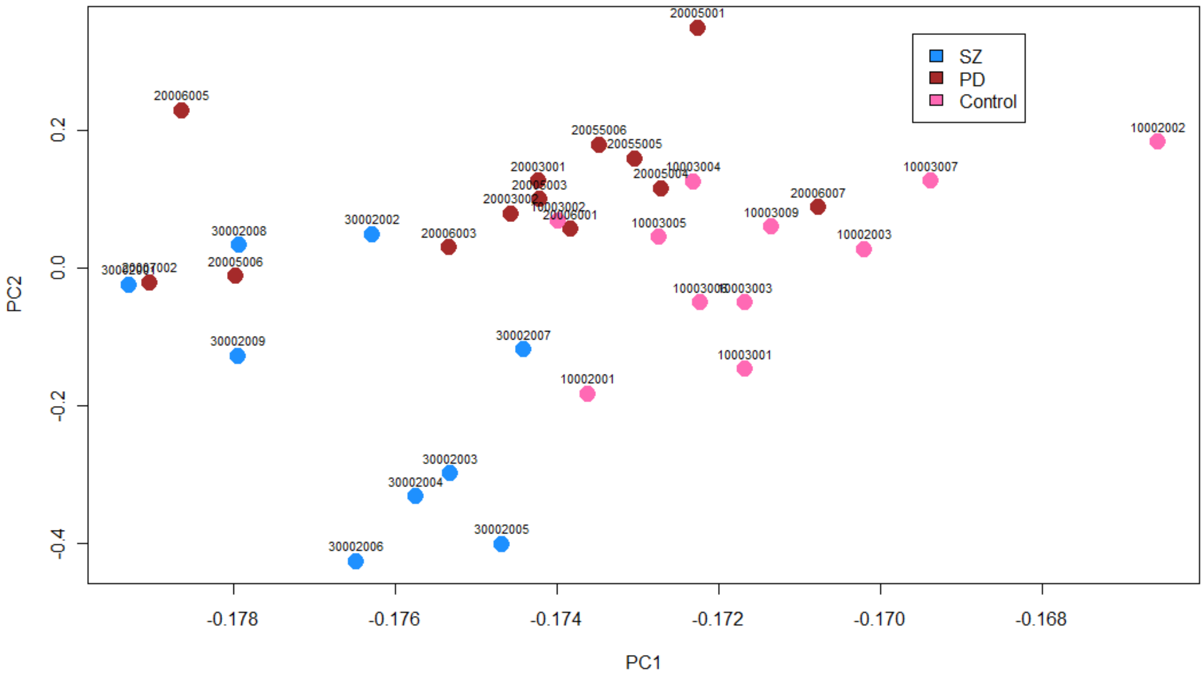

PCA on Just Background Corrected Data Set, Tech Reps Averaged + Detected Probes Only

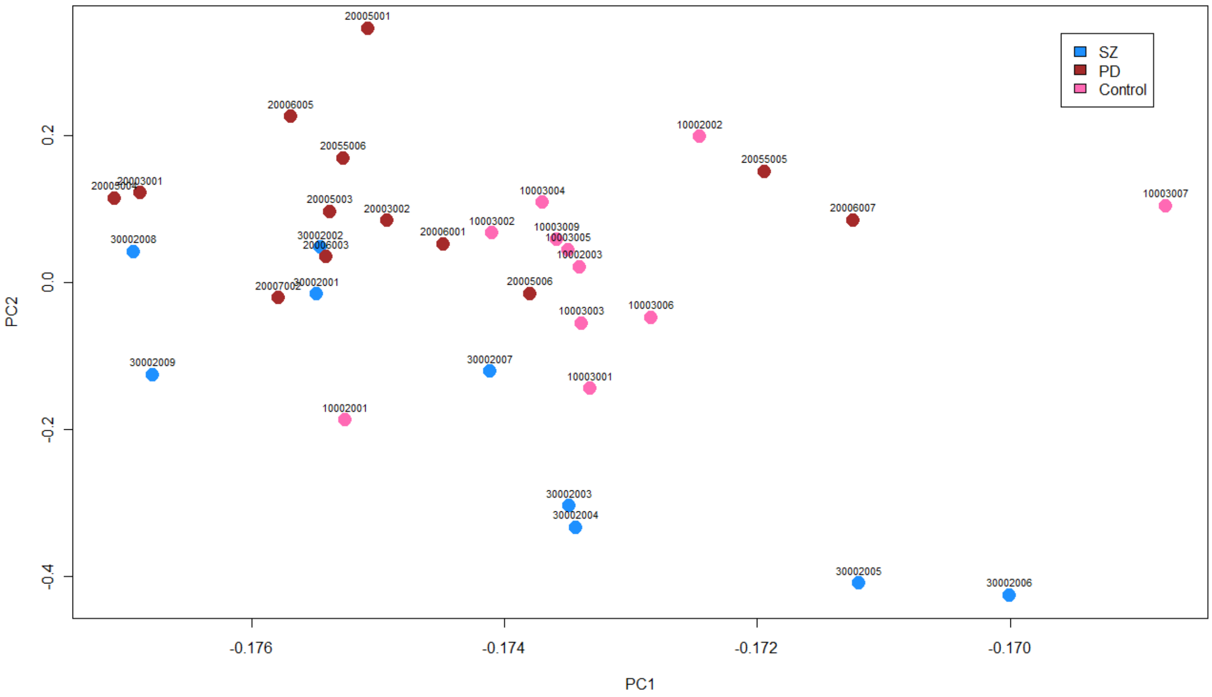

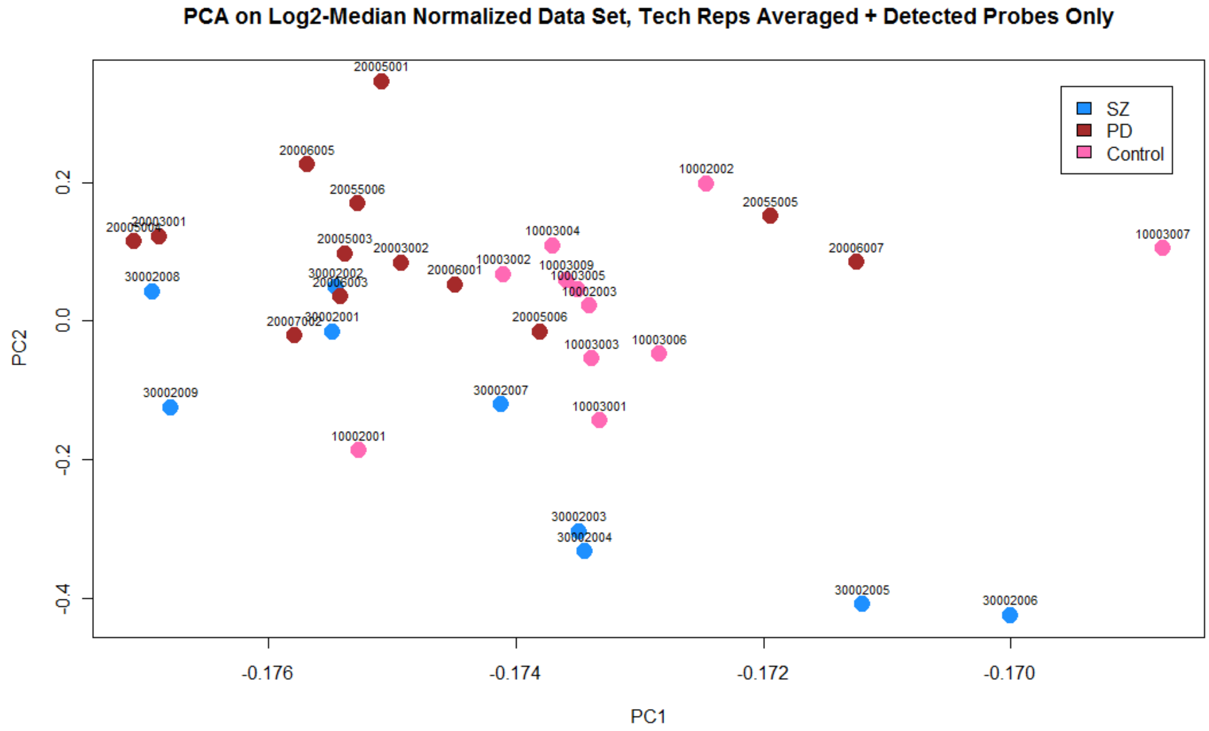
PCA on Log2-Median Normalized Data Set, Tech Reps Averaged + Detected Probes Only
SZ
PD
Control
PC1
PC2
-0.176
-0.174
-0.172
-0.170
0.2
0.0
-0.2
-0.4

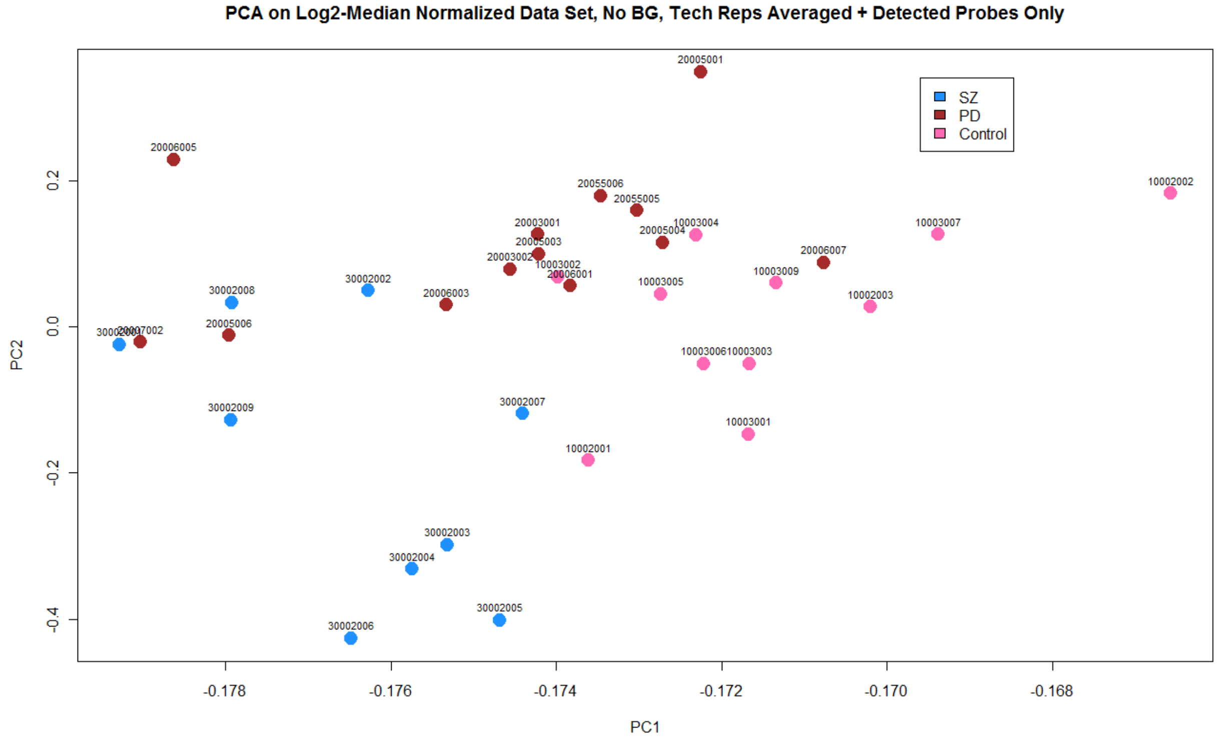
PCA on Log2-Median Normalized Data Set, No BG, Tech Reps Averaged + Detected Probes Only
SZ
PD
Control
PC1
PC2
-0.178
-0.176
-0.174
-0.172
-0.170
-0.168
0.2
0.0
-0.2
-0.4

PCA on Quantile Normalized Data Set, No BG, Tech Reps Averaged + Detected Probes Only

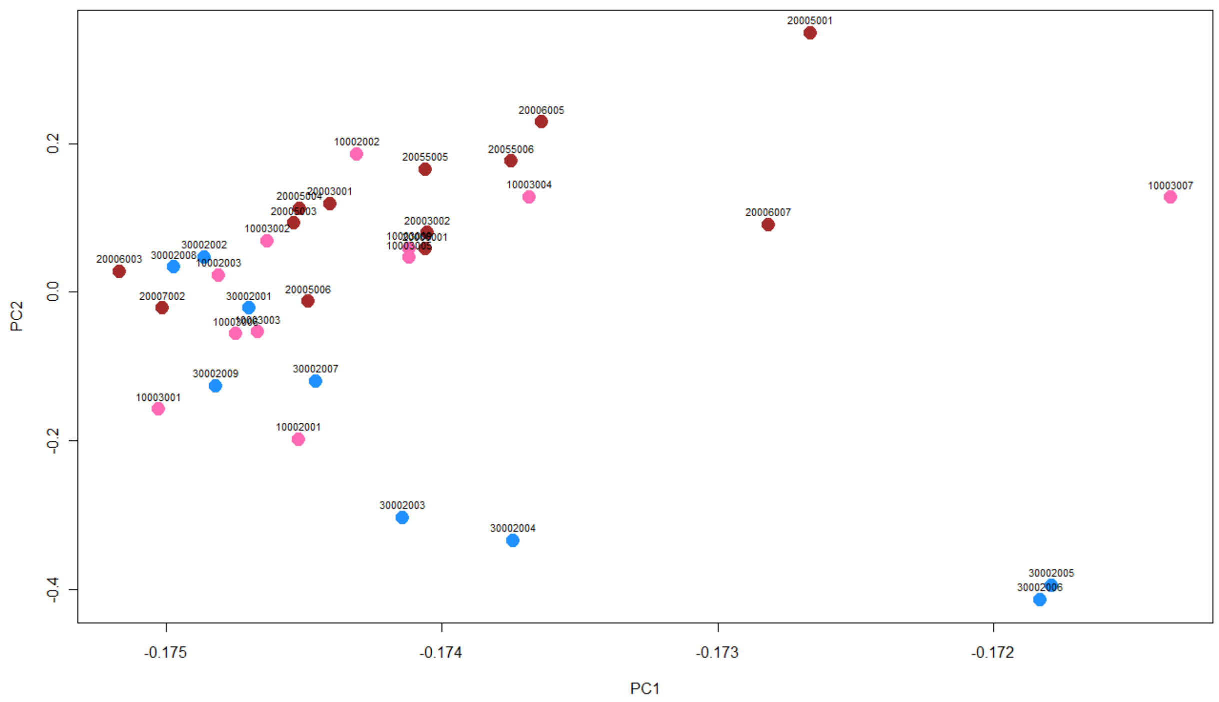

PCA on Quantile Normalized Data Set, Tech Reps Averaged + Detected Probes Only

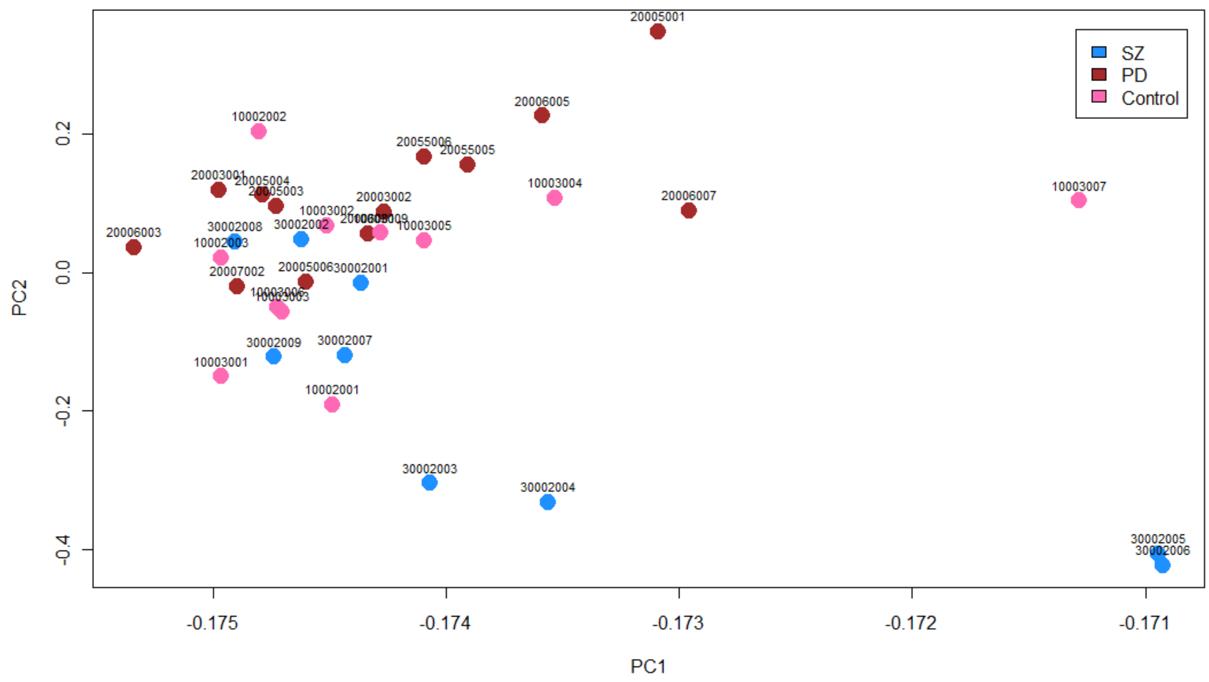

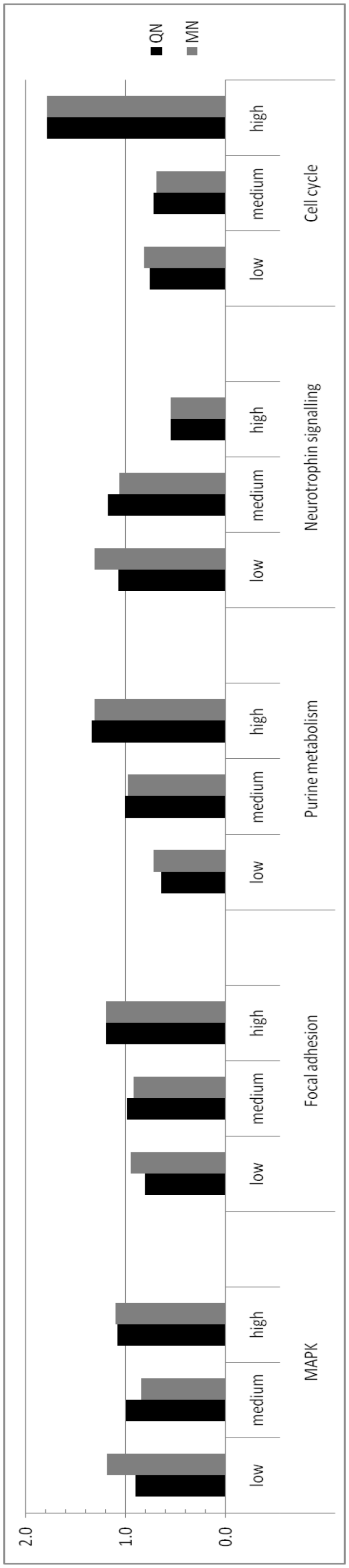
QN
MN
2.0
1.0
0.0
low
medium
high
MAPK
low
medium
high
Focal adhesion
low
medium
high
Purine metabolism
low
medium
high
Neurotrophin signalling
low
medium
high
Cell cycle

We next compared variance across the top 5 attract pathways using median or quantile normalization for the control hONS samples (previous page). The overall patterns of variance were very similar, and the pathways with significantly skewed variance were apparent despite the data transformation strategy used. This is also illustrated by the comparison of a wider selection of data transformation strategies on the MAPK pathway variance (below).

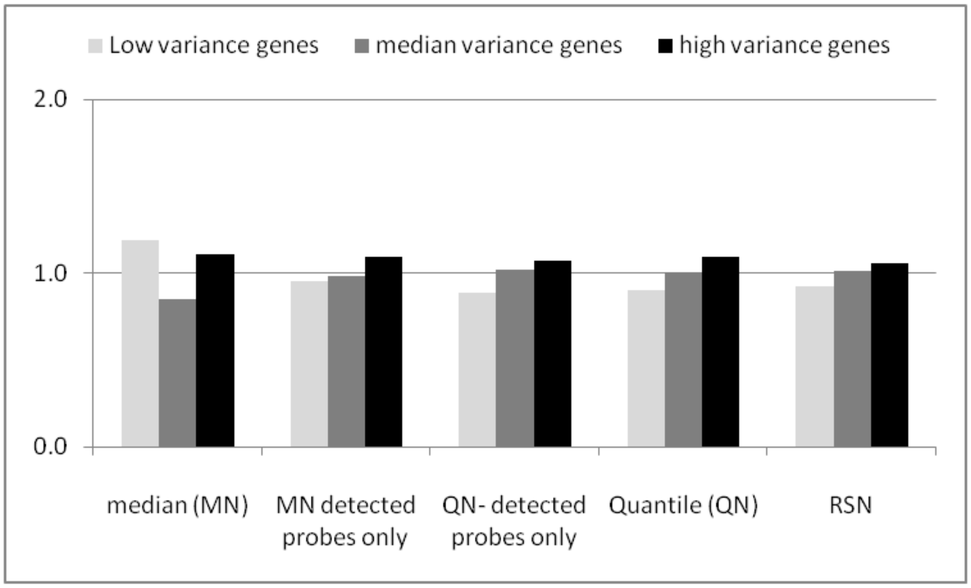

Given that the normalization strategies overall provided similar variance distributions for the control datasets, we next compared median normalization with quantile normalization for the disease cohorts. Quantile normalization consistently best reflected the variance patterns seen in the raw data (next page).

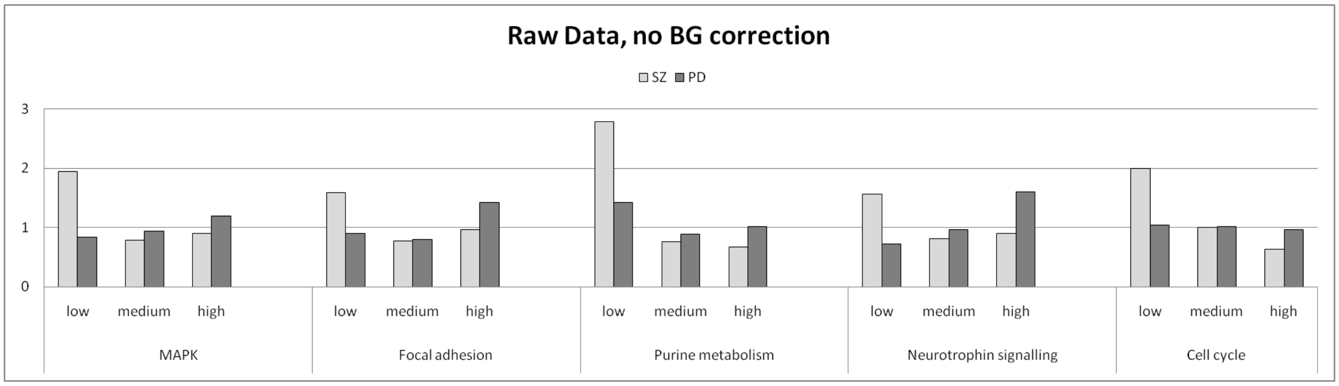

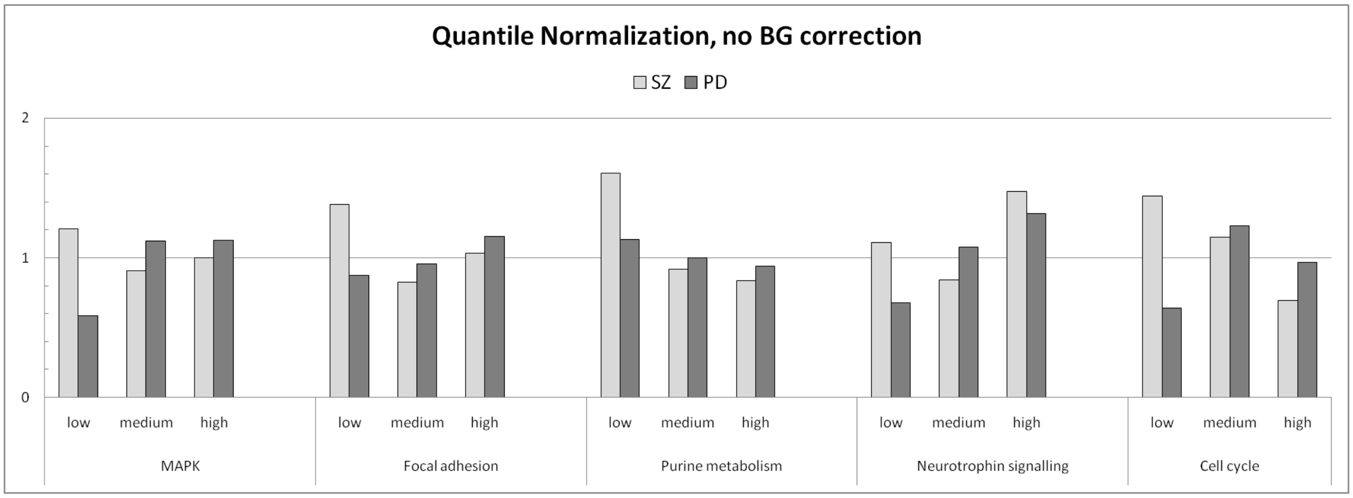

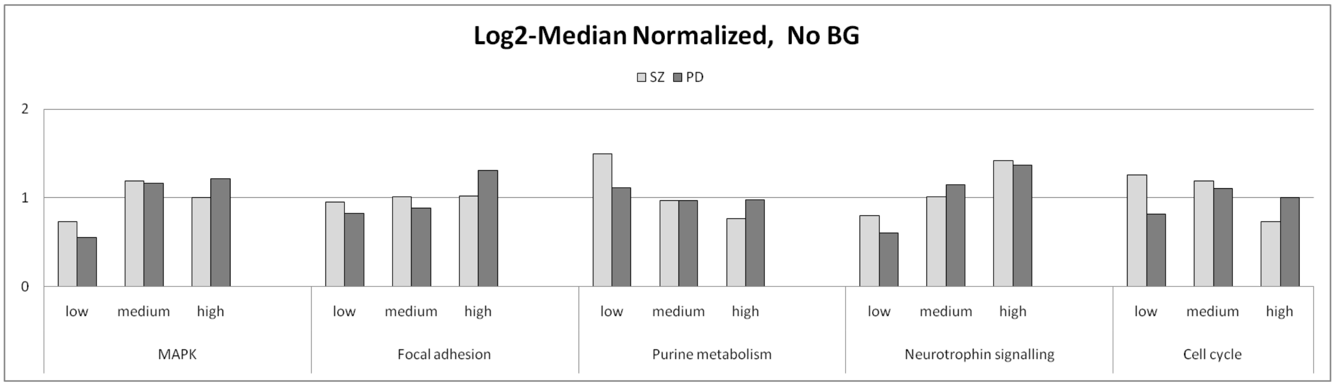

The impact of these data transformations was also examined on the hONS and fibroblasts comparisons.

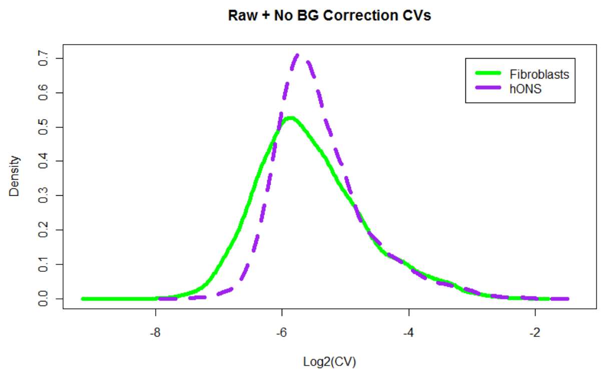
Raw + No BG Correction CVs
Density
Log2(CV)
Fibroblasts
hONS

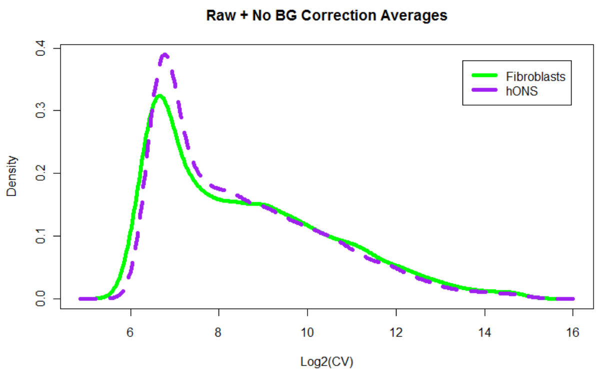
Raw + No BG Correction Averages
Density
Log2(CV)
Fibroblasts
hONS

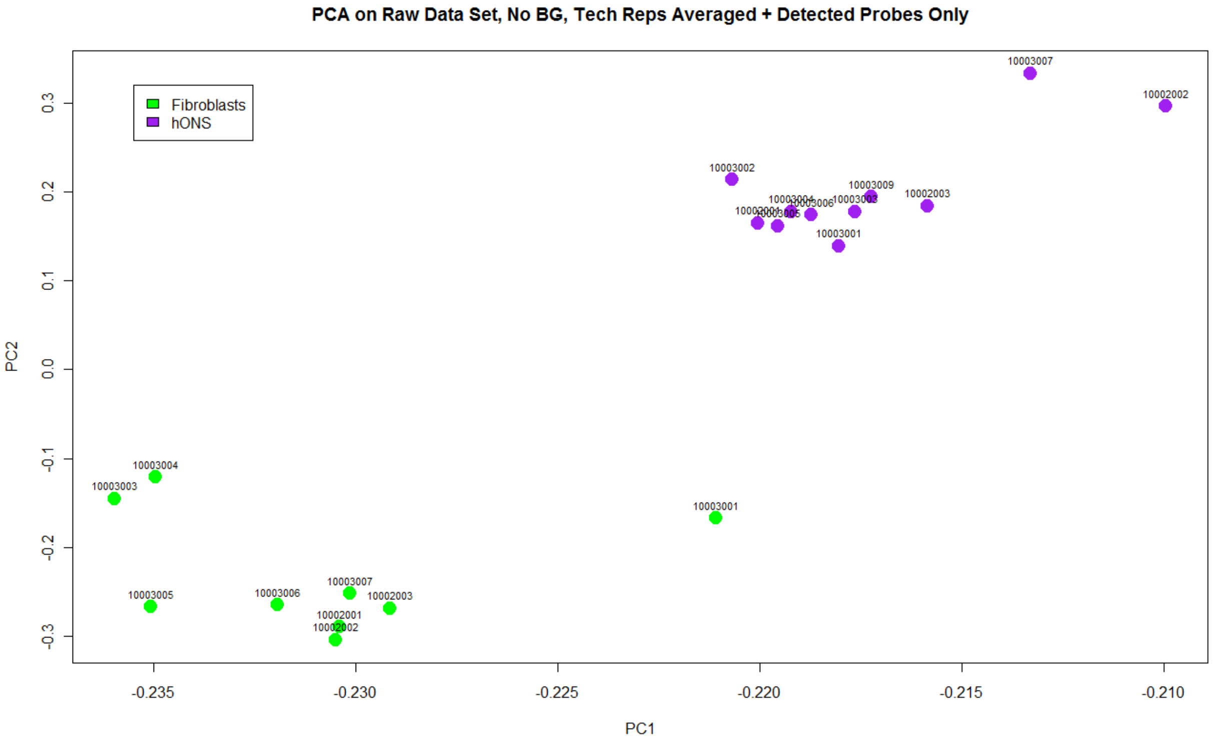
PCA on Raw Data Set, No BG, Tech Reps Averaged + Detected Probes Only
PC1
PC2
Fibroblasts
hONS

LogMedian Normalized + No BG Correction CVs

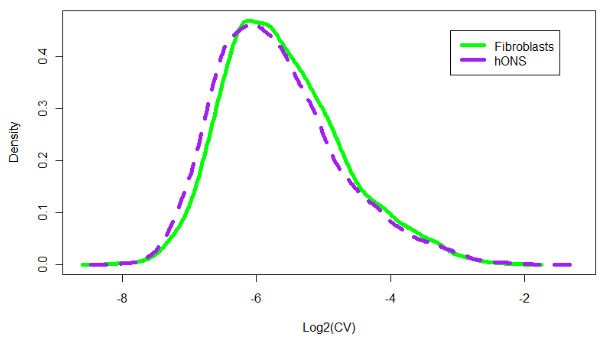

LogMedian Normalized + No BG Correction Averages

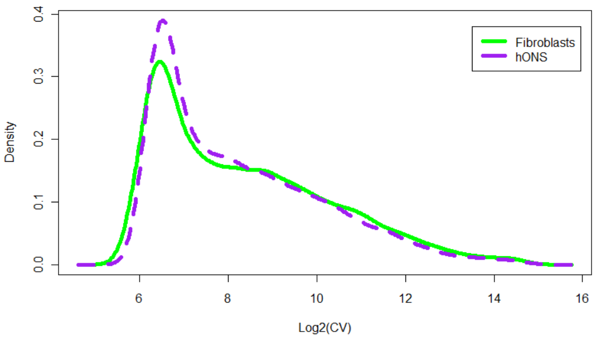

LogMedian Normalized (detected probes only) + No BG Correction CVs

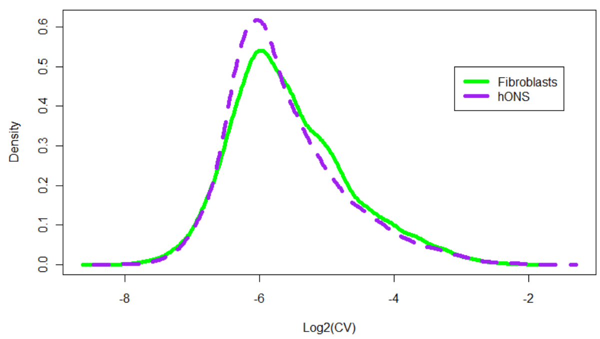

LogMedian Normalized (detected probes only) + No BG Correction Averages

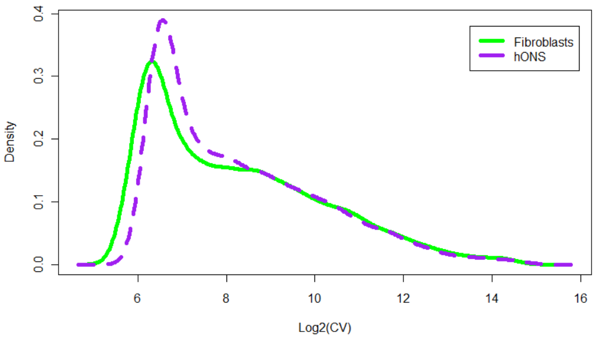

PCA on Log2-Median Normalized Data Set, No BG, Tech Reps Averaged + Detected Probes Only

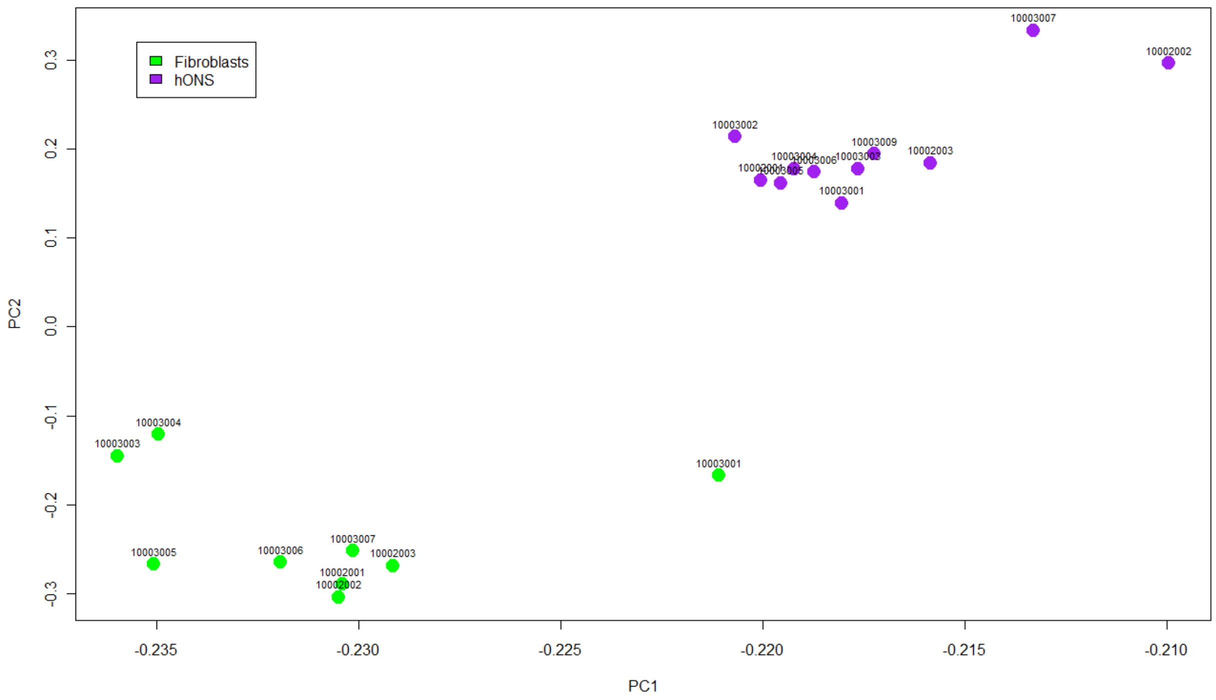

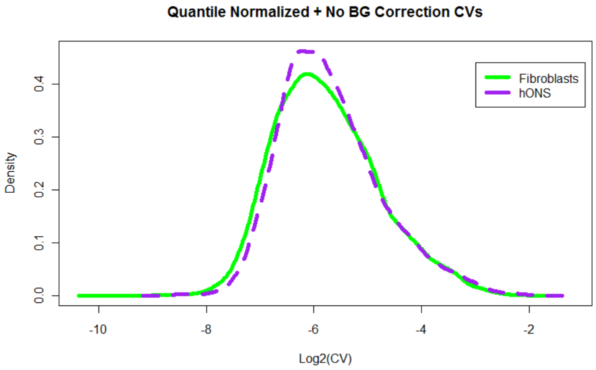
Quantile Normalized + No BG Correction CVs
Density
Log2(CV)
Fibroblasts
hONS
0.0
0.1
0.2
0.3
0.4
-10
-8
-6
-4
-2

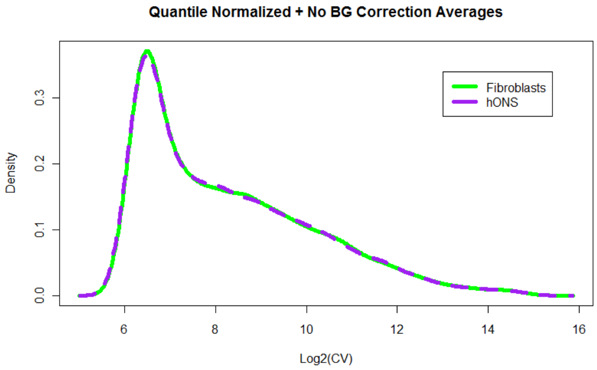
Quantile Normalized + No BG Correction Averages
Density
Log2(CV)
Fibroblasts
hONS
0.0
0.1
0.2
0.3
6
8
10
12
14
16

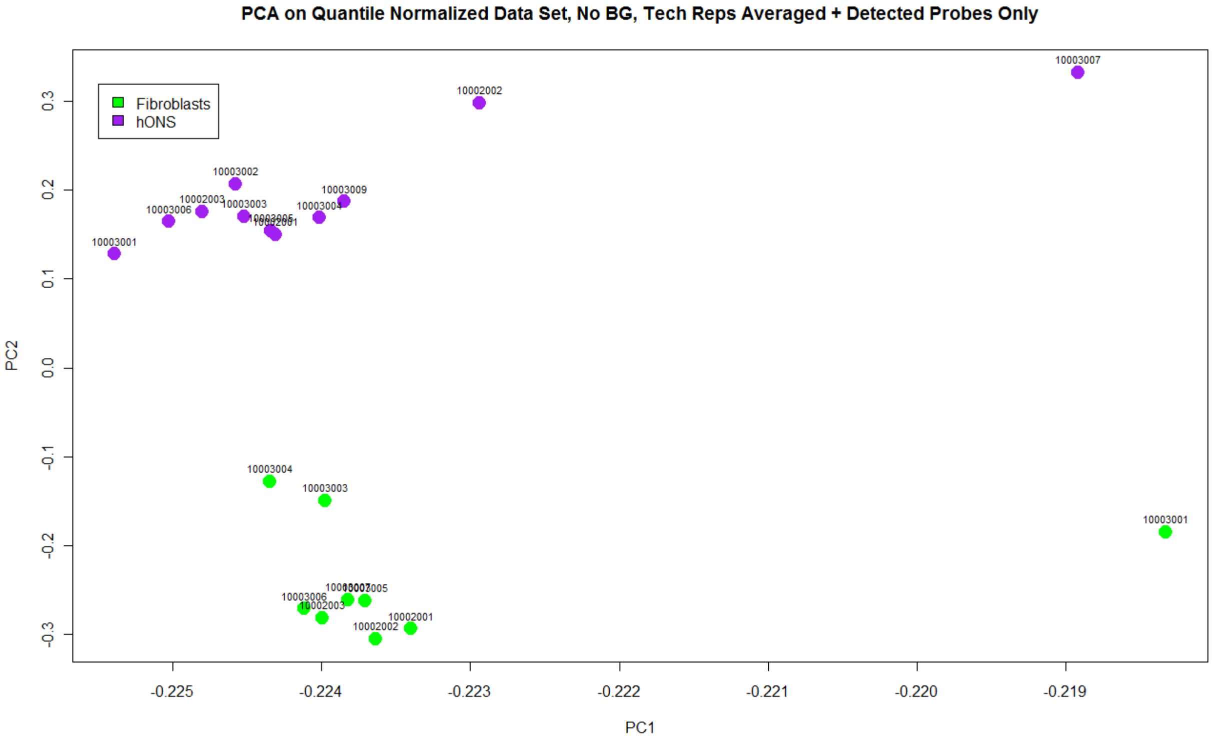
PCA on Quantile Normalized Data Set, No BG, Tech Reps Averaged + Detected Probes Only
Fibroblasts
hONS
PC1
PC2
10003007
10002002
10003002
10003009
10002003
10003003
10003006
10003004
10003001
10003004
10003003
10003001
10003006
10002003
10002001
10002002
-0.3
-0.2
-0.1
0.0
0.1
0.2
0.3
-0.225
-0.224
-0.223
-0.222
-0.221
-0.220
-0.219

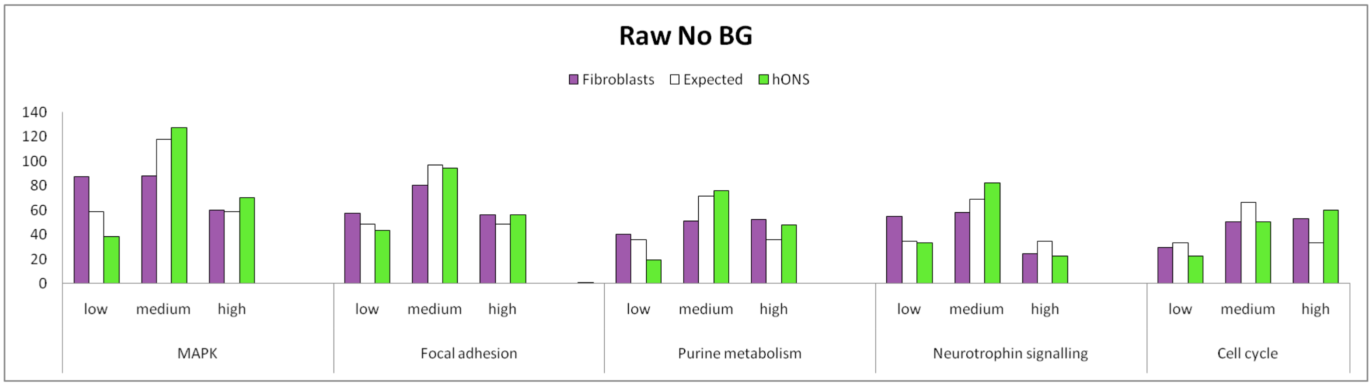
Raw No BG
Fibroblasts
Expected
hONS
140
120
100
80
60
40
20
0
low
medium
high
MAPK
Focal adhesion
Purine metabolism
Neurotrophin signalling
Cell cycle

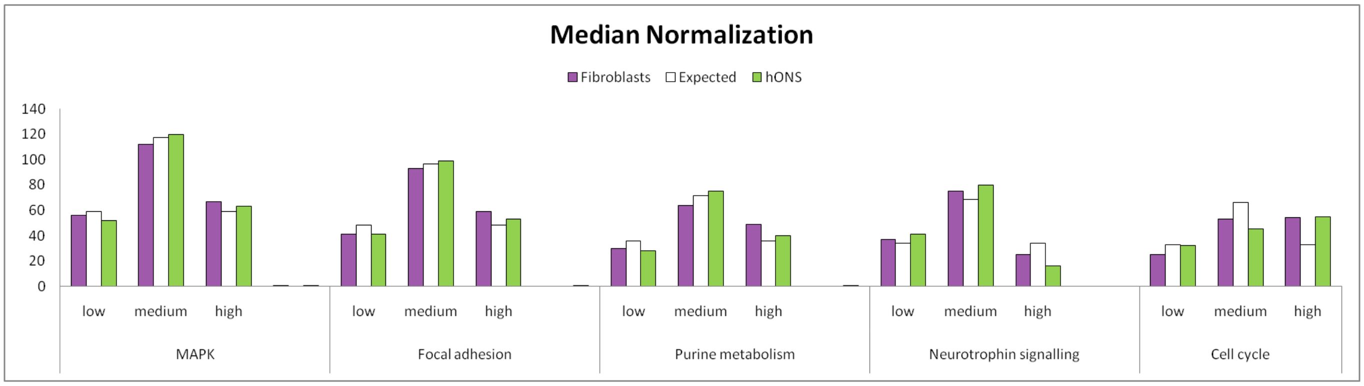
Median Normalization
Fibroblasts
Expected
hONS
140
120
100
80
60
40
20
0
low
medium
high
MAPK
Focal adhesion
Purine metabolism
Neurotrophin signalling
Cell cycle

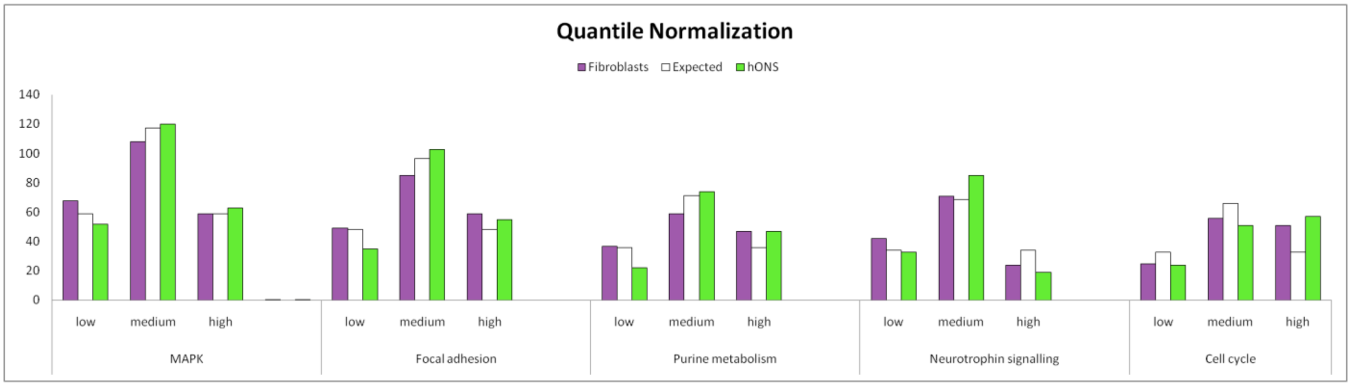
Quantile Normalization
Fibroblasts
Expected
hONS
140
120
100
80
60
40
20
0
low
medium
high
MAPK
Focal adhesion
Purine metabolism
Neurotrophin signalling
Cell cycle
