# Supplementary material for: Variance of Gene Expression Identifies Altered Network Constraints in Neurological Disease
Source: PLoS Genet. 2011 Aug 11;7(8):e1002207. doi: 10.1371/journal.pgen.1002207 (PMC3154954; doi:10.1371/journal.pgen.1002207)

Supplementary Discussion 2: Comparing Variability Count Distributions Across Disease States

There are 216 genes annotated to the MAPK signalling pathway. We observe these genes assorting into high, medium and low variance groups according to the following gene counts:

	Control group	Schizophrenia	Parkinson's disease
High variance	64	64	72
Medium variance	118	107	132
Low variance	53	64	31

Genes are classified as high, medium and low variance based on their position in the expression variance distribution. The regions of low, medium and high variance are defined by the upper 25th and bottom 25th percentiles.

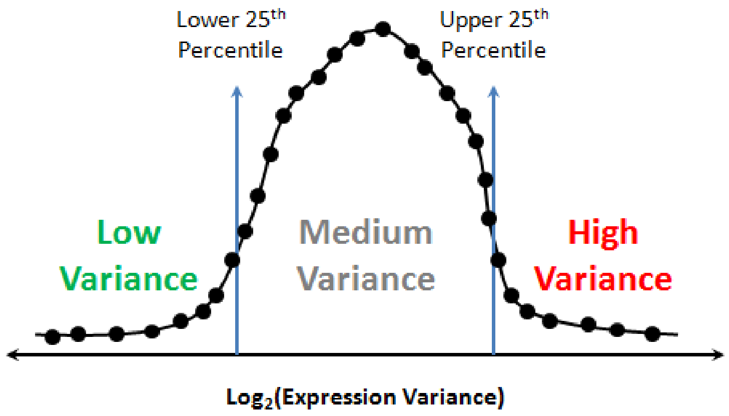

Therefore by design, for the MAPK signalling pathway, we expect to see the following gene counts just by chance:

	Expected Distribution
High variance	54
Medium variance	108
Low variance	54

The significance of deviation between the counts observed for the donor groups versus what we expect to see by chance can be assessed using a Chi-squared test with 2 degrees of freedom.

	Schizophrenia vs expected	Parkinson's disease vs expected	Control vs expected
P-value	0.191	0.003	0.247

Similarly, we can evaluate the significance of deviation between the gene counts from the disease states versus the control group.

	Schizophrenia vs Control	Parkinson's disease vs control
P-value	0.156	0.00003

Testing the Robustness of the Disease-Specific Variance Profiles Observed

We permutated the sample labels amongst the three donor groups, selecting at random (without replacement) 9, 9 and 13 labels to represent the permutated SZ, Control, and PD groups respectively. For each permutated data set, we calculated the number of high, medium and low variance genes using the original 25% cut-offs for the CV values. Using a Chi-square test, we assessed the deviation between the counts in the disease group and the control group. 1000 permutations were performed, and the 1000 P-values were adjusted for multiple testing using the Benjamini-Hochberg correction method.

We note in the permutated data sets, we observed no differences between the three donor groups in the number of genes mapping to the high, medium, and low variance classes.

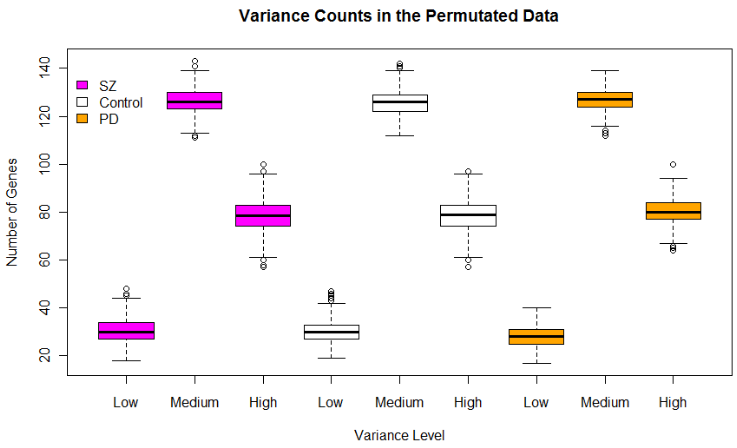

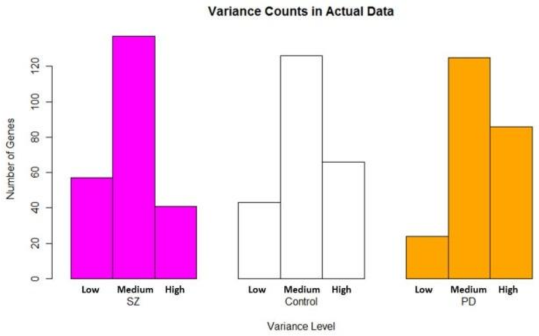

The 1000 adjusted P-values were approximately uniformly distributed in the SZ vs. Control comparison, or slightly skewed towards larger P-values for the PD vs. Control comparison. We note that the probability of observing a more extreme adjusted P-value than the one we observed for SZ vs. Control, by chance, is 1/1000 and for PD vs. Control is less than 1/1000.

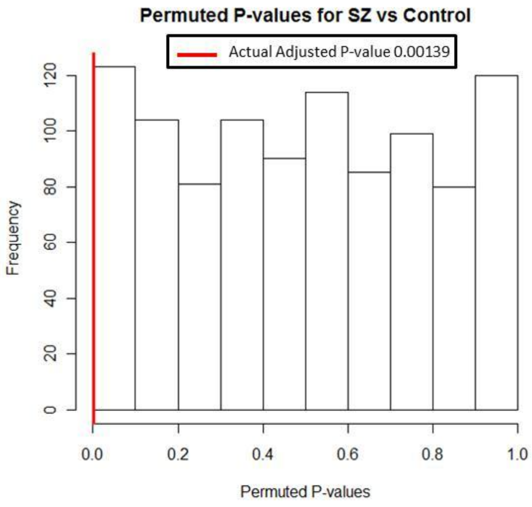

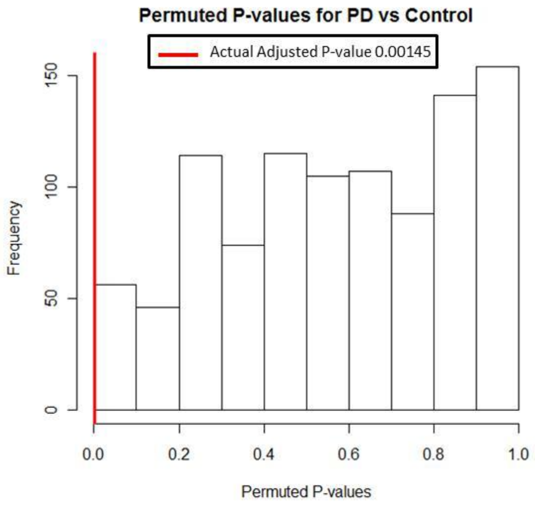

Supplement: Text S2 — Supplementary discussion on comparing gene counts between donor groups. (PDF) [file pgen.1002207.s004.pdf]
