# Supplementary material for: Variance of Gene Expression Identifies Altered Network Constraints in Neurological Disease
Source: PLoS Genet. 2011 Aug 11;7(8):e1002207. doi: 10.1371/journal.pgen.1002207 (PMC3154954; doi:10.1371/journal.pgen.1002207)
Supplement: Text S3 — Discussion of intra-donor variance patterns from additional donors with independently derived duplicate biopsies, and comparison to the between-donor variance patterns. (PDF) [file pgen.1002207.s005.pdf]

Supplementary Discussion: Analyzing Intra- versus Inter-individual Variability Patterns

We assessed the contribution of technical variation to expression variance by using the gene expression data collected for four donors who each had two independently derived biopsies. These biopsies were themselves replicated twice, giving rise to four arrays per individual. We constructed intra-individual variation distributions for each of the four individuals, and compared this to the distributions observed for the donor groups in both hONS stem cell lines and fibroblasts lines.

The intra- and inter-individual data sets were each separately summarized using BeadExplorer, and background corrected and quantile normalized using the lumi package. A detection filter was applied where probes had to satisfy detection P-values < 0.01 in at least 75% of the samples within the same group. (For the intra-individual data set, a group corresponded to the four replicate samples for the same individual). Only data from the overlapping set of probes between the intra- and inter-individual detected probes were retained for further analysis; this overlap consisted of 6809 probes.

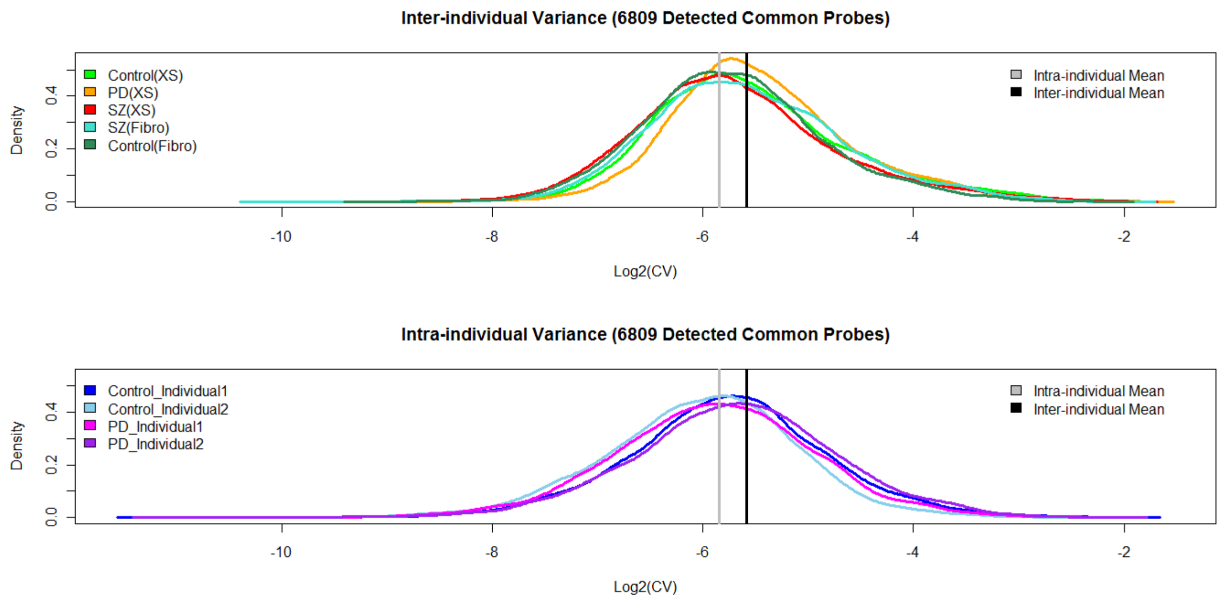

On average, the intra-individual values (mean = -5.845) are smaller than the inter-individual values (mean = -5.588), confirming the observation that the technical variation in this data set is less than biological variation associated with the donor groups. Although the difference between the inter-individual and intra-individual means is small, if the number of individuals with replicated measurements available was to grow, we would expect this difference to grow.

Controlling for Sample Size

By fixing the number of samples contributing to the inter-individual variance to match the sample size for the intra-individual variance (n=4), we can assess the effect sample size has on calculating the variance.

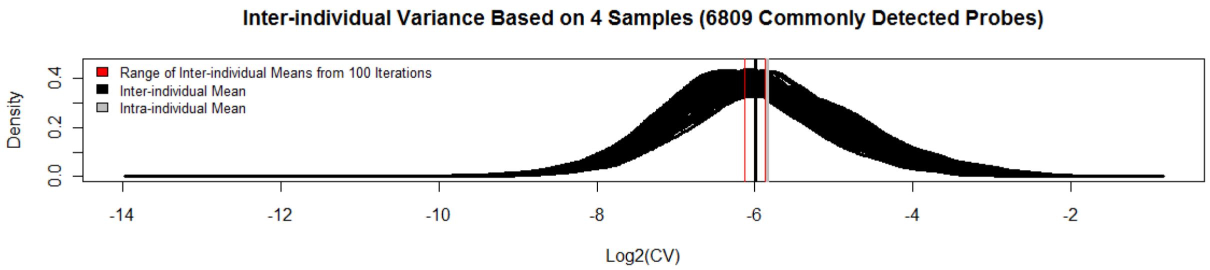

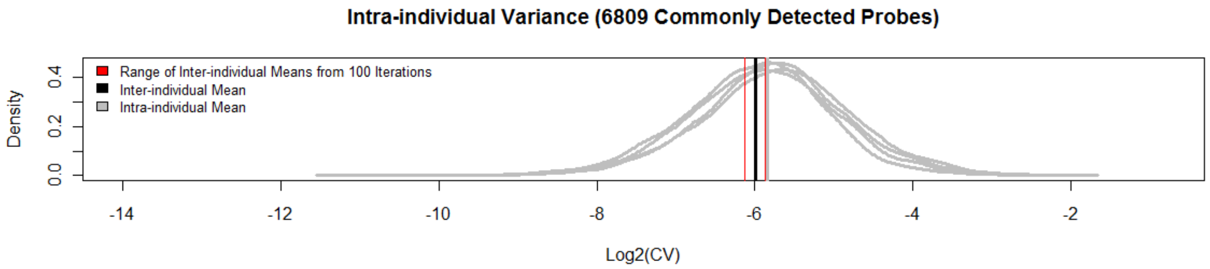

We observe a very small difference between the inter- and intra-individual means (-5.993 versus -5.845, respectively; with the inter-individual values ranging between -6.120 and -5.872). This suggests that reducing the sample size to fixed numbers has very little effect on the variance distributions we are seeing since we do not see a dramatic change when smaller sample sizes are used to calculate the intra- and inter-individual variance distributions.
