# Supplementary material for: Variance of Gene Expression Identifies Altered Network Constraints in Neurological Disease
Source: PLoS Genet. 2011 Aug 11;7(8):e1002207. doi: 10.1371/journal.pgen.1002207 (PMC3154954; doi:10.1371/journal.pgen.1002207)
Supplement: Text S4 — Supplementary tutorial on calculating the degree distribution. (PDF) [file pgen.1002207.s006.pdf]

Supplementary Tutorial 1: Testing for Differences in Degree Distributions

For each donor group, the set of high and low expression variance genes are each associated with their own connectivity or degree distribution within a signalling network. The degree distribution characterizes the proportion of genes with the same degree or connectivity.

For example, the high and low variance degree distributions for the control group for the MAPK signalling network were:

<u>Degree distribution for High Variance Genes in Control Group</u>

Degree	0	1	2	3	4	5	6	8	9	10	11
% of Genes	0.169811	0.075472	0.264151	0.113208	0.056604	0.056604	0.056604	0.018868	0.037736	0.018868	0.037736

Degree	13	15	16	17
% of Genes	0.037736	0.018868	0.018868	0.018868

<u>Degree distribution for Low Variances Genes in the Control Group</u>

Degree	1	2	3	4	5	6	8	9	10	11	13
% of Genes	0.078947	0.052632	0.131579	0.052632	0.052632	0.026316	0.131579	0.052632	0.052632	0.026316	0.026316

Degree	15	17	18	19	20	21	23	26	29	30
% of Genes	0.026316	0.026316	0.026316	0.026316	0.078947	0.026316	0.026316	0.026316	0.026316	0.026316

Since the two distributions span slightly different ranges of degree number, we focus only on testing the deviation between the two distributions for the genes with the same degree.

Under the null hypothesis that both distributions were generated from the same parameters, we designate the high variance group as the observed distribution, and use this to calculate the expected distribution for the low variance group.

For the MAPK network, there were 38 low variance genes for the control group, so the predicted or expected distribution is:

Degree	1	2	3	4	5	6	8	9	10	11	13	15	17
Number of Genes	2.867925	10.03774	4.301887	2.150943	2.150943	2.150943	0.716981	1.433962	0.716981	1.433962	1.433962	0.716981	0.716981

Degree	1	2	3	4	5	6	8	9	10	11	13	15	17
Number of Genes	3	2	5	2	2	1	5	2	2	1	1	1	1

The test statistic measuring the deviation between distributions is given as:

$$X = \sum_{j=0}^{14} \frac{(Expected_j - Observed_j)^2}{Expected_j} \sim \chi_9^2$$

and follows a Chi-squared distribution with the number of different degrees – 1; so for this example there are 9 degrees of freedom. The P-value associated with this test statistic is 0.000351; the difference between the degree distributions for the high and low variance genes is highly significant.

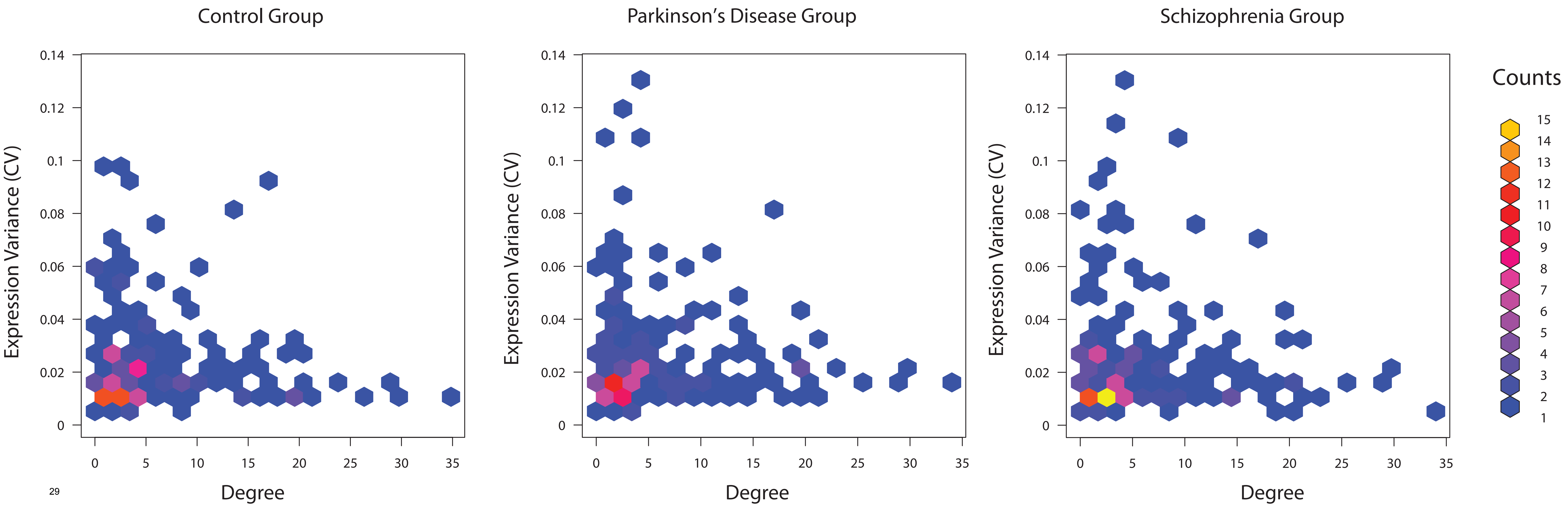
Control Group
Parkinson's Disease Group
Schizophrenia Group
Expression Variance (CV)
Degree
0.14
0.12
0.1
0.08
0.06
0.04
0.02
0
0
5
10
15
20
25
30
35
Counts
15
14
13
12
11
10
9
8
7
6
5
4
3
2
1
